# Components of Endocannabinoid Signaling System Are Expressed in the Perinatal Mouse Cerebellum and Required for Its Normal Development

**DOI:** 10.1523/ENEURO.0471-19.2020

**Published:** 2020-04-21

**Authors:** Luis Ricardo Martinez, Kylie Caroline Black, Brynna Tellas Webb, Alexandria Bell, Shawyon Kevin Baygani, Tristen Jay Mier, Luis Dominguez, Ken Mackie, Anna Kalinovsky

**Affiliations:** 1 The Gill Center for Biomolecular Science, Program in Neuroscience, Department of Psychological and Brain Sciences, Indiana University, Bloomington, 47405 IN

**Keywords:** ataxia, cannabinoid, CB1, cerebellum, coordination, development

## Abstract

Endocannabinoid (eCB) signaling system (ECS), encompassing cannabinoid receptors and enzymes involved in the synthesis and degradation of the endogenous cannabinoid signaling lipids, is highly expressed in the cerebellar cortex of adult humans and rodents. In addition to their well-established role in neuromodulation, eCBs have been shown to play key roles in aspects of neurodevelopment in the fore- and mid-brain, including neurogenesis, cell migration, and synapse specification. However, little is known about the role of ECS in cerebellar development. In this study, we conducted immunohistochemical characterization of ECS components through key stages of cerebellar development in mice using antibodies for 2-arachidonoylglycerol (2-AG) synthetizing and degrading enzymes and the major brain cannabinoid receptor, cannabinoid receptor 1 (CB1), in combination with cerebellar cell markers. Our results reveal a temporally, spatially, and cytologically dynamic pattern of expression. Production, receptor binding, and degradation of eCBs are tightly controlled, thus localization of eCB receptors and the complementary cannabinoid signaling machinery determines the direction, duration, and ultimately the outcome of eCB signaling. To gain insights into the role of eCB signaling in cerebellar development, we characterized gross anatomy of cerebellar midvermis in CB1 knock-out (CB1 KO) mice, as well as their performance in cerebellar-influenced motor tasks. Our results show persistent and selective anatomic and behavioral alterations in CB1 KOs. Consequently, the insights gained from this study lay down the foundation for investigating specific cellular and molecular mechanisms regulated by eCB signaling during cerebellar development.

## Visual Abstract

**Figure:**
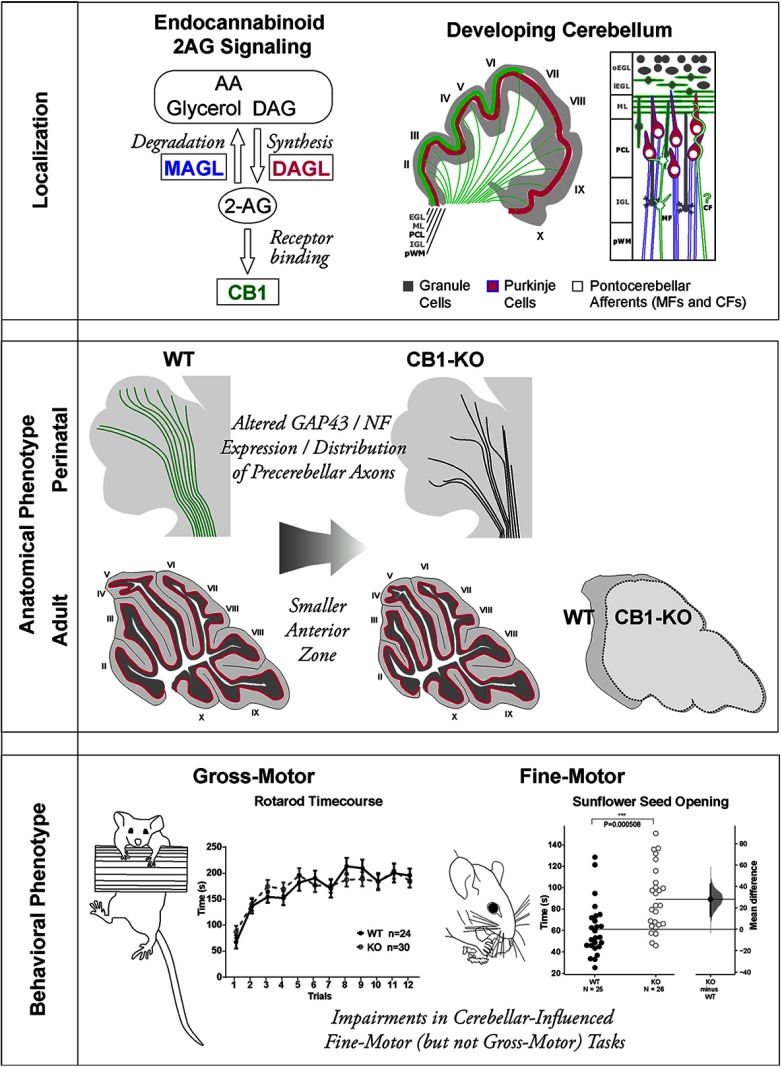

## Significance Statement

In this study, we show that components of the endocannabinoid (eCB) signaling system (ECS) are prominently expressed in the perinatal [embryonic day (E)17.5 to postnatal day (P)14] mouse hindbrain. Our comprehensive characterization highlights developmentally dynamic and spatially restricted expression of cannabinoid receptor 1 (CB1), prominent at birth in pontocerebellar axons, and later in migrating and differentiating anterior vermis granule cells (GCs). We identify the role of Purkinje cells (PCs) in the regulation of eCB 2-arachidonoylglycerol (2-AG) availability, since they express both catabolic and anabolic enzymes diacylglycerol lipase α (DAGLα) and monoacylglycerol lipase (MAGL). Furthermore, we demonstrate a requirement for eCB signaling in the regulation of pontocerebellar axon distribution and of postnatal cerebellar growth. CB1 knock-outs (KOs) exhibit impairments in cerebellar-influenced fine-motor, but not gross-motor behaviors. Together, these results illuminate a previously unrecognized role of eCB signaling in the regulation of cerebellar development and function.

## Introduction

Previous studies have shown robust and dynamic expression of the endocannabinoid (eCB) signaling system (ECS) during forebrain development, and its requirement in regulation of multiple neurodevelopmental aspects (Berghuis et al., 2007; Mulder et al., 2008; Vitalis et al., 2008; Wu et al., 2010; Oudin et al., 2011; Díaz-Alonso et al., 2012). However, very little is known about the dynamics of ECS expression, or the role that eCB signaling plays, in the developing hindbrain. Thus, the ubiquity of the regulatory roles of eCB signaling in neurodevelopment remains an open question. Furthermore, despite clear evidence of the key role that eCB signaling plays in the regulation of cerebellar synaptic plasticity (Carey et al., 2011), the role of eCB signaling in cerebellar development has not yet been investigated. In this study, we undertook detailed immunohistochemical characterization of ECS localization in the perinatal hindbrain followed by characterization of anatomic and behavioral consequences of genetic cannabinoid receptor 1 (CB1) ablation.

### Cerebellar development

The intricate cerebellar cytoarchitecture emerges during a protracted developmental period (for review, see Sathyanesan et al., 2019). Shortly after the initiation of the cerebellar primordium, granule cell (GC) precursors populate the outer-most layer of the developing cerebellum forming the external GC layer (EGL), a secondary proliferative zone dedicated to GC production, which persists until postnatal day 21 (P21) in mice. Under the EGL, differentiating Purkinje cells (PCs) are located in the PC layer (PCL). Over the first four postnatal weeks, postmitotic GCs migrate inwards and settle underneath the PCL to seed the inner GC layer (IGL), while their axons form parallel fibers (PFs) and begin to form synapses with dendrites of PCs in the developing molecular layer (ML) above PCL. As the IGL expands, PCs rearrange to form a monolayer (around P7). Over the following two postnatal weeks the EGL is gradually depleted and disappears, and by P28 cerebellar cytoarchitecture and synaptic connectivity mostly achieves its mature configuration. On the gross anatomic level, proliferation and maturation of the cerebellar cell types is accompanied by cerebellar expansion and increasing complexity and refinement of cerebellar anatomic and functional subdivisions.

### Neurodevelopmental disorders associated with cerebellar lesions

Lesions in the developing cerebella are linked to syndromes encompassing motor, cognitive, and emotional deregulation (Koziol et al., 2014). The specific nature of the deficits depends on their location within the cerebellum. Hence, traumatic and malignant lesions in the anterior cerebellar vermis and paravermis lead to deficits in fluid and accurate execution of complex movements involving the face, head, hands, trunk, and feet, often characterized as dysarthria, ataxia, or dysmetria (Konczak et al., 2005; Schoch et al., 2006; Ilg et al., 2008; Basson and Wingate, 2013). On the other hand, posterior fossa syndrome, a common complication in pediatric patients following surgery that involves the posterior cerebellar vermis and deep cerebellar structures, is associated with mutism, executive control and affective disturbances, apathy, alterations in attention, anxiety, and compulsive behaviors (De Smet et al., 2009).

### eCB signaling in cerebellar development

CB1 is the primary cannabinoid receptor that is highly expressed in the adult cerebella of both rodents and humans (Herkenham et al., 1990, 1991b; Kawamura et al., 2006; Carey et al., 2011). 2-Arachidonoylglycerol (2-AG) is the most abundant eCB found in the cerebellum, and diacylglycerol lipase α (DAGLα) is one of the major biosynthetic enzymes contributing to its production (Yoshida et al., 2006; Tanimura et al., 2010). The spatial and temporal specificity of eCB signaling is also regulated by localization and activity of eCB degradation enzymes. Monoacylglycerol lipase (MAGL) is a key enzyme involved in 2-AG hydrolysis (Blankman et al., 2007).

In order to address whether CB1, DAGLα, and MAGL are present in the developing pontocerebellar system, we used previously verified antibodies in combination with cerebellar cell type markers at key ages during perinatal development: embryonic day 17.5 (E17.5), P0, P3, P5, P8, P10, P12. To elucidate requirements for eCB signaling in cerebellar development, we characterized gross anatomic parameters throughout a developmental time course revealing a persistent reduction in midvermal cerebellar area, accompanied by selective impairments in cerebellar-controlled motor behaviors in young-adult mice.

## Materials and Methods

### Mice

All mice used in this study were maintained on an outbred CD1 genetic background.

The CD1 IGS stock was initially acquired from Charles River (https://www.criver.com/products-services/find-model/cd-1r-igs-mouse?region=3611) and a breeding colony was maintained in the vivarium with 12/12 h light/dark cycle under conditions stipulated by the Institutional Animal Care and Use Committee. *cb1* −/− mice [CB1 knock-out (KO)] used in this study were generated by (Ledent et al., 1999), acquired from Charles River France and maintained in our facility by HET x HET crosses. Genetic diversity was maintained by replenishing CD1 founders from Charles River annually. Mice of both sexes were used for all experiments. Since we observed no significant differences between sexes in expression, anatomy or behavior, data are shown for the two sexes combined for all experiments.

### Tissue preparation

For embryonic tissue collection pregnant dams were killed by isoflurane inhalation, embryos were harvested and whole heads were immersion-fixed in 4% paraformaldehyde (PFA) at 4°C overnight, followed by brain dissection and additional immersion postfixation in 4% PFA for 4 h at 4°C. All postnatal tissue was fixed by transcardial perfusion with PBS followed by 4% PFA. Dissected brains were postfixed in 4% PFA at 4°C for 1 h. All brains were stored at 4°C in 0.2% sodium azide PBS (PBS-Az) and sectioned at 70 μm using a Leica VT 1000S vibrating microtome.

#### Antibodies and immunohistochemistry

Immunohistochemistry was performed on free-floating 70-μm tissue sections. Tissue sections were washed three times in 1× PBS and incubated in BSA blocking buffer (5% BSA/0.5% Triton X-100/PBS). Primary antibodies were applied overnight at 4°C in BSA blocking buffer. Subsequently, slides were washed three times in 1× PBS and secondary antibodies (Alexa Flour 488, 594, 647 from Jackson ImmunoResearch) were applied at 4°C overnight (1:600 in BSA blocking buffer). DraQ5 (Cell Signaling) was used (at 1:5000 in PBS) to visualize cell nuclei. Slides were coverslipped using Fluoromount G (SouthernBiotech). Details regarding antibodies against CB1, DAGLα, and MAGL (including target epitopes, prior publications and working dilutions) are listed in Table 1. All information related to cerebellar cell marker antibodies is listed in Table 2.

**Table 1:** Primary antibodies against CB1, DAGLα, and MAGL: target epitopes, prior publications, RRIDs, and working dilutions

eCB signaling machinery					
**Antibody**	**Immunogen**	**Source**	**RRID**	**Working** **dilution**	**Reference**
Rabbit anti-CB1	Synthetic peptide, aa 443–473	ImmunoGenes, polyclonal	AB_2813823	1:300	Dudok et al. (2015)
Rabbit anti-CB1 L15	CB1-GST fusion protein, aa 460–473 of rat CB1	Custom made, polyclonal	AB_2315250	1:300	Bodor et al. (2005)
Guinea pig anti-CB1 L15	Last 15 aa on L-term of human CB1	Custom made, polyclonal	AB_2813824	1:600	Berghuis et al. (2007)
Guinea pig anti-DAGLα	aa 790–908 of rat DGLα	Custom made, polyclonal	AB_2813825	1:600	Katona et al. (2006)
Rabbit anti-MAGL	GST fusion protein, aa 172–206 of mouse MAGL	Custom made, polyclonal	AB_2813826	1:600	Straiker et al. (2009)

**Table 2:** Commercially available primary antibodies used to identify cerebellar cell types: immunogens, sources, RRIDs, and working dilutions

Cerebellar cell type markers				
**Antibody**	**Immunogen**	**Source**	**RRID**	**Working dilution**
Mouse anti-SMI312 (pan-axonal neurofilament cocktail)	SMI 312 is directed against highly phosphorylated axonal epitopes of neurofilaments	BioLegend, #837904, monoclonal	AB_2566782	1:1000
Rabbit anti-Pax-6	Peptide QVPGSEPDMSQYWPRLQ from the C terminus of mouse Pax-6 protein	BioLegend, clone poly-19013, #901301, polyclonal	AB_2565003	1:600 After antigen retrieval
Goat anti-RORα	Peptide mapping at the C terminus of RORα1 of human origin	Santa Cruz, sc-6062, polyclonal	AB_655755	1:100 at E17.5–P0 1:300 at P3–P20
Mouse anti- TuJ-1 (neuron-specific beta-III tubulin)	Monoclonal mouse IgG_2A_ clone #TuJ-1; detects mammalian and chicken neuron-specific beta–III tubulin; raised against rat brain-derived microtubules	R&D Systems, #MAB1195, monoclonal	AB_357520	1:1000 at 1 DIV
Mouse anti-Calb	Monoclonal anti-calbindin D-28k is a mouse IgG1 produced by hybridization of mouse myeloma cells with spleen cells from mice immunized with calbindin D-28k purified from chicken gut	Swant, #300, monoclonal	AB_10000347	1:500 at P0–P5 1:1000 at P6–P15 1:3000 at P16–adult
Rabbit anti-Calb	Against recombinant rat calbindin D-28k	Swant, #CB38, polyclonal	AB_2721225

#### GC culture

Preparation of GC cultures followed a previously published protocol (Manzini et al., 2006; Lee et al., 2009), which produces >90% pure and developmental stage synchronized GCs. In short, cerebella were dissected from P5 mice in L-15 media (Invitrogen), dissociated by incubation in 15 μg/ml DNAse1 (Sigma) 0.15% Trypsin (Invitrogen) at 37°C, followed by trituration. Cells were pelleted and resuspended in supplemented Neurobasal (Invitrogen) media, plated on poly-D-ornithine**/**laminin (Cultex) coated eight-well LabTech (Nunc) dishes. Cultures were grown in 5% CO_2_ at 37°C for 24h in supplemented Neurobasal media. Cultured cells were fixed by underlying culture media with 4% PFA, and incubating in 4% PFA for 20 min at room temperature. Staining was performed following the same protocol as for brain slices.

#### Microscopy

Images were acquired using Leica LAS software on a Leica TSC SP5 confocal microscope (Leica Microsystems) in the IUB LMIC facility. Tiled images were acquired with 10× objective and 5- to 10-μm Z-stacks collected at 1-μm Z intervals. High-resolution images were acquired with a 63× objective as 20-μm Z-stacks consisting of 0.4-μm-thick optical sections. Images are displayed as collapsed max-projection Z-stacks. Figures were prepared in Adobe Photoshop and Adobe Illustrator.

#### Tissue sectioning and Nissl staining

Twenty-micrometer sagittal sections were serially collected using a cryostat (Leica). Following PBS and dH_2_O washes, sections were incubated in 0.1% Cresyl violet solution, followed by dehydration, clearing in xylene, and mounting in Permount. Images of whole cerebellar sections were obtained using 4× objective and mosaics with an Applied Precision DeltaVision Nikon microscope and Motic EasyScan at IUB Imaging Facility. Figures were prepared in Adobe Photoshop and Adobe Illustrator.

#### Morphologic analysis

Areas of total cerebellar midvermis, lobes I–III, and lobes IX–X were traced from midsagittal sections using the free-hand tool in FiJi (https://imagej.nih.gov/nih-image/manual/tools.html).

#### Statistical Analysis (Fig. 10; Table 3)

Estimation stats multi two groups, was performed in https://www.estimationstats.com/#/analyze/multi. Data were collected from 106 animals of both sexes over a P3, P5, P12, and two-month time course. Numbers of animals and litters analyzed for each genotype and age group are detailed in Tables 3, 4. We assume a normal distribution of data points. The statistical hypothesis tested the magnitude of differences in area means between WTs and KOs; *p* values were evaluated by two-sided Mann–Whitney test. Effect sizes and uncertainty (bootstrapped intervals) are shown in Figures 10, 11 and in Tables 3, 4.

**Table 3:** Statistical table for Figure 10

Differences in area means between WT and KO							
	**Age**	**WT (control); *n* = animals;** ***N* = litters**	**KO (test);** ***n* = animals;** ***N* = litters**	**Difference**	**95% CI of** **difference**	***p* value** **Mann–** **Whitney**	*<0.05 **<0.01 ***<0.005 ****<0.0001
Total Midvermal Area	P3	*n* = 6 *N* = 2	*n* = 8 *N* = 1	0.22046525	0.1005809 to 0.33800371	0.02386844	*
Total Midvermal Area	P5	*n* = 23 *N* = 10	*n* = 19 *N* = 9	–0.5345564	–1.1207783 to –0.0094494	0.08802591	ns
Total Midvermal Area	P12	*n* = 8 *N* = 2	*n* = 8 *N* = 2	–0.8547641	–1.3264183 to –0.3690421	0.01008169	*
Total Midvermal Area	2 months	*n* = 23 *N* = 6	*n* = 11 *N* = 5	–1.544694	–2.3539983 to –0.929924	0.00040931	***
lobes I–III	P3	*n* = 6 *N* = 2	*n* = 8 *N* = 1	0.00060315	–0.0444497 to 0.04175221	0.94853252	ns
lobes I–III	P5	*n* = 23 *N* = 10	*n* = 19 *N* = 9	–0.3107878	–0.4960554 to –0.1219811	0.00145207	***
lobes I–III	P12	*n* = 8 *N* = 2	*n* = 8 *N* = 2	–0.5471343	–0.7196807 to 0.4179097	0.00093911	***
lobes I–III	2 months	*n* = 23 *N* = 6	*n* = 11 *N* = 5	–0.9491027	–1.1671892 to –0.7593015	1.1904E-06	****
lobes IX–X	2 months	*n* = 21 *N* = 6	*n* = 11 *N* = 5	–0.384772	–0.5777334 to –0.2250031	0.00064456	***
Differences in ratio of subregions over total midvermal areas between WT and KO							
I–III/total	P3	*n* = 6 *N* = 2	*n* = 8 *N* = 1	–0.0465324	–0.0774791 to –0.0181734	0.02386844	*
I–III/total	P5	*n* = 23 *N* = 10	*n* = 19 *N* = 9	–0.0725127	–0.0951794 to –0.0456856	2.4409E-05	****
I–III/total	P12	*n* = 8 *N* = 2	*n* = 8 *N* = 2	–0.0728148	–0.0866715 to –0.0584468	0.00093911	***
I–III/total	2 months	*n* = 21 *N* = 6	*n* = 11 *N* = 5	–0.0391941	–0.0483767 to –0.0289259	1.275E-05	****
IX–X/total	2 months	*n* = 21 *N* = 6	*n* = 11 *N* = 5	–0.0046574	–0.012602 to 0.0042559	0.40473443	ns

**Table 4:** Statistical table for Figure 11

Difference in latency to open sunflower seeds between WT and KO										
										
**Condition**	***n*; *N***	**Number of trials** **per animal**	**Mean**	**Difference** **betweenmeans**	**SE ofdifference**	**95% CI ofdifference**	***p* value,Mann–Whitney**	*<0.05,**<0.01,***<0.005, ****<0.0001		
WT*n* = animals,*N* = litters	*n* = 25;*N* = 10	2–5	61	27.9297959	30.1743641	12.0735231to42.2478872	0.00050806	***		
KO*n* = animals,*N* = litters	*n* = 26;*N* = 9	2–5	88							
Difference in latency to fall from rotarod between WT and KO										
										
**Condition**	***n*; *N***	**Number of trials(3 per day)**	**Mean**	**Differencebetweenmeans**	**SE ofdifference**	**95% CI ofdifference**	***p* value (mixedeffects analysis)**	*<0.05, **<0.01,***<0.005,****<0.0001	**Area under thecurve (all trials)**	**WT/KO ratio of areas underthe curve**
WT*n* = animals,*N* = litters	*n* = 24;*N* = 10	12	170.7	–1.24	13.97	–29.28 to26.80	0.9296	ns	1775	0.986
KO*n* = animals,*N* = litters	*n* = 30;*N* = 10	12	172.0						1799.5	

### Behavior

#### Rotarod

Two-month-old mice were placed on an accelerating rotarod (UGO Basile) which accelerated from 4 to 40 rpm over a 5-min period. The latency until the mouse fell off was recorded. Mice were given three trials over four consecutive days with a maximum time of 300 s (5 min) per trial and given 15 min to recover between trials.

#### Statistical analysis

Data were collected from a total of 54 animals (sexes combined) over a time course of 12 trials (three per day). We assume a normal distribution of data points. The statistical hypothesis of equivalent latency to fall between genotypes was evaluated by Tukey multiple comparison, and by comparing areas under the curve for all trials. Statistical analysis was performed in GraphPad Prism 8. The details on the number of mice in each group, mean and *p* values are provided in Figure 11 and in Table 4. In addition, improvement in rotarod performance was evaluated by fitting linear regression curves over the first six trials, and by comparing differences in slopes between genotypes. The relationship between rotarod performance and limb grip strength was also evaluated.

#### Seed opening

Two-month-old mice were food deprived overnight (12 h), and then placed in the testing cage with four seeds. For each seed, the time was recorded from the first contact with the seed until the mouse stopped interacting with the seed. Only trials in which the seed was at least 75% opened/consumed were included in the analysis. Data for each mouse is an average of two to five trials.

#### Statistical analysis

Data were collected from a total of 51 animals (sexes combined). We assume a normal distribution of data points. The differences in latency to open a seed were evaluated by two-independent-groups mean difference in Estimation Stats (https://www.estimationstats.com/#/analyze/two-independent-group); *p* values were evaluated by two-sided Mann–Whitney test. Effect sizes and uncertainty (bootstrapped intervals) are shown in Figure 11 and in Table 4.

## Results

### CB1 is prominently expressed in long-range axons in the brainstem and the cerebellum at E17.5 and during the first postnatal week

**Figure 1.**
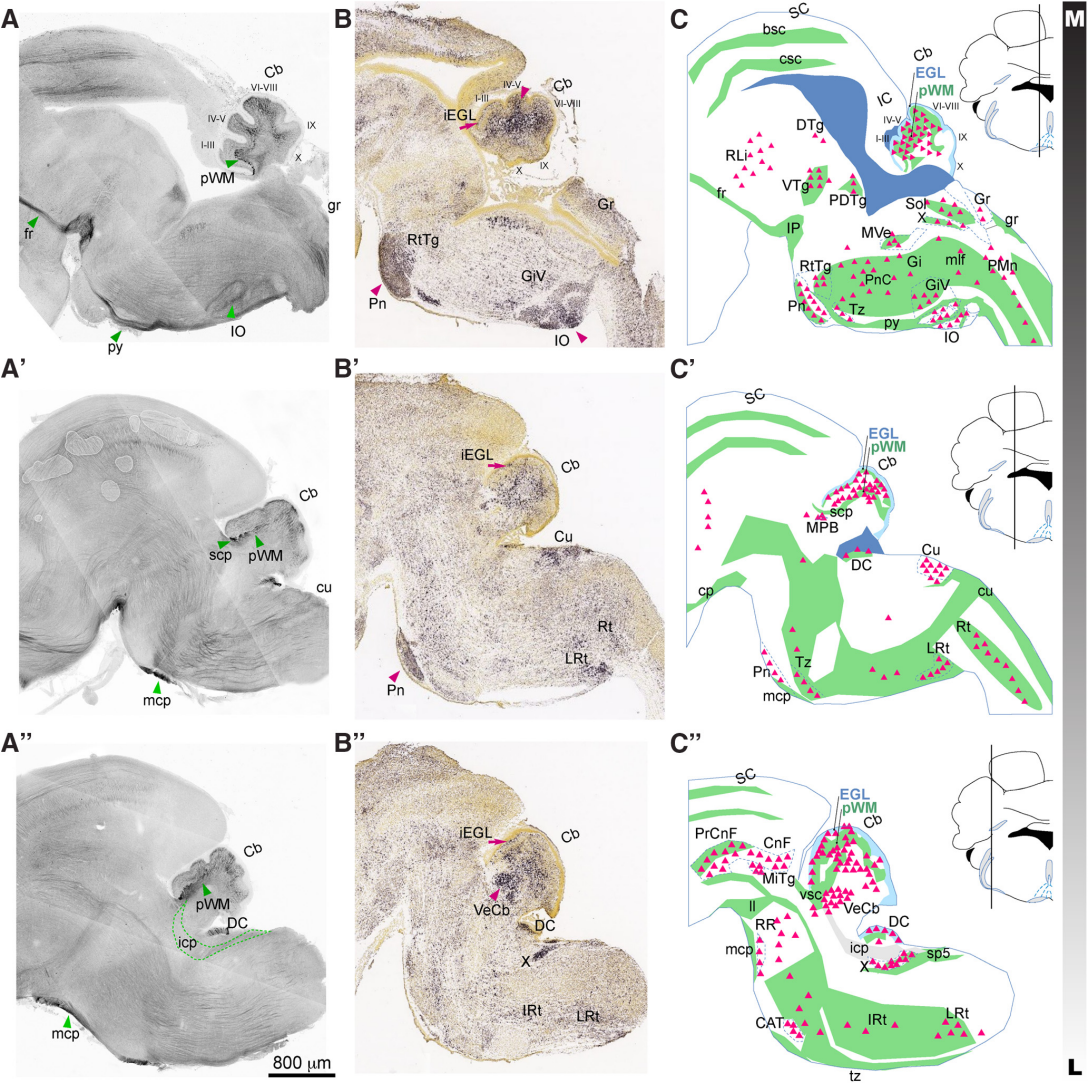
At birth, CB1 is prominently expressed in axon tracts in hindbrain. ***A–A”***, Immunolocalization of CB1 in sagittal hindbrain sections spanning cerebellar vermis and paravermis at P0 shows robust expression in developing axons, including long-range axon tracts in the brainstem and in the fibers cruising through the inner regions of the developing cerebellar cortex. Immediately relevant for the developing pontocerebellar system, CB1 immunolocalization is seen in mcp and scp [mcp (***A”***) and scp (***A’***), highlighted by green arrowheads], and in axons filling pWM (***A–A”***, green arrowheads). CB1 expression in icp (***A”***, green dotted outline) is very weak or absent. ***B–B”***, Sagittal sections showing localization of CB1 mRNA in the hindbrain at E18 from the Allen Brain Atlas Developmental Mouse database (2008 Allen Institute for Brain Science. Allen Developing Mouse Brain Atlas, available from http://developingmouse.brain-map.org/experiment/show/100024806). Notably for pontocerebellar development, CB1 mRNA expression is prominent both in the Pn (***B***, maroon arrowhead) and the IO (***B***, maroon arrowhead), among many other brainstem nuclei which project axons to the cerebellum (marked by maroon triangles and anatomic abbreviations in summary diagrams in panels ***C–C”***). In the cerebellum, CB1 mRNA is most prominent in deeper layers (***B***, maroon arrowhead). CB1 mRNA positive region spans developing IGL and pWM, marked in summary diagram in ***C***. CB1 mRNA expression is also prominent in vestibulocerebellar nucleus (VeCb; ***B”***, maroon arrowhead). In addition, restricted regions of the iEGL (***B–B”***, maroon arrow) show thin layer of CB1-positive cells. ***C–C”***, Traced outlines of sections shown in *A*, summarizing perinatal distribution of CB1 expression relative to anatomic landmarks. Green shapes mark CB1 immunolocalization in axon tracts as shown in *A*, red triangles mark brain regions with robust CB1 mRNA expression as shown in ***B***. Lines in coronal insets represent mediolateral plane of sectioning. Anatomical locations are adapted from Franklin and Paxinos (2008) and from Paxinos et al. (2007). bsc, brachium of superior colliculus; CAT, nucleus of the central acoustic tract; CB, cerebellum; CnF, cuneiform nucleus; cp, cerebral peduncle; csc, commissure of superior colliculus; Cu, cuneate nucleus; DC, dorsal cochlear nucleus; DTg, dorsal tegmental nucleus; fr, fasciculus retroflexus; Gi, gigantocellular reticular nucleus; GiV, gigantocellular reticular nucleus, ventral part; g, gracile fasciculus; Gr, gracile nucleus; icp, inferior cerebellar peduncle; IC, inferior colliculus; IO, inferior olivary nucleus; IP, interpeduncular nucleus; IRt, intermediate reticular nucleus; ll, lateral lemniscus; LRt, lateral reticular nucleus; mcp, middle cerebellar peduncle; mcp, middle cerebellar peduncle; MiTg, microcellular tegmental nucleus; mlf, medial longitudinal fasciculus; MPB, medial parabrachial nucleus; MVe, medial vestibular nucleus, magnocellular part; PDTg, posterodorsal tegmental nucleus; PMn, paramedian reticular nucleus; Pn, pontine nucleus; PnC, pontine reticular nucleus, caudal part; PrCnF, precuneiform area; py, pyramidal tract; RLi, rostral linear nucleus (midbrain); RR, retrobulbar nucleus; Rt, reticular nucleus; RtTg, reticulotegmental nucleus of the pons; SC, superior colliculus; scp, superior cerebellar peduncle; Sol, solitary nucleus; sp5, spinal trigeminal tract; Tz, nucleus of the trapezoid body; tz, trapezoid body; VeCb, vestibulocerebellar nucleus; vsc, ventral spinocerebellar tract; VTg, ventral tegmental nucleus; X, nucleus *X*, dorsal nucleus of the vagus nerve.

**Figure 2.**
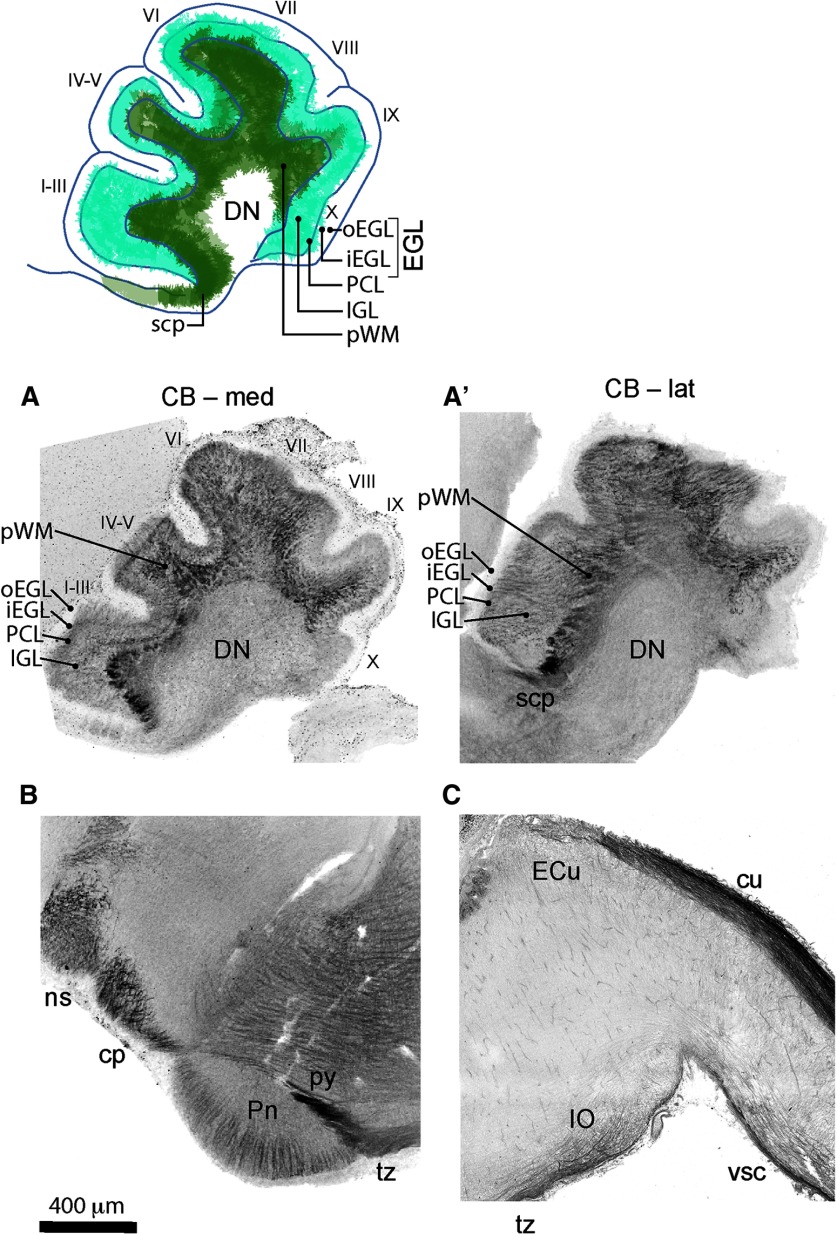
Zoomed-in view of CB1 distribution is the brainstem and the cerebellum at P0. Top row: diagram showing distribution of CB1 staining relative to the layers of the developing cerebellum (superimposed on top of the tracing of medial cerebellar section). ***A***, ***A’***, Thick CB1-positive fibers are prominent in the scp and pWM (darker green in the diagram). Thinner CB1-positive structures (lighter and more diffuse staining) span iEGL through PCL and IGL (turquoise in the diagram). CB1 staining is very week or absent from the oEGL, and DN. ***B***, CB1-positive fibers are radially oriented in Pn. CB1 is prominently expressed within multiple axon tracts visible in this section through the brainstem (ns, cp, py, tz). ***C***, CB1-positive fibers can be seen within the territory of the IO, and running through the cuneate tract (cu). Anatomical locations are adapted from Franklin and Paxinos (2008) and from Paxinos et al. (2007). CB, cerebellum; cp, cerebral peduncle; cu, cuneate fasciculus; DN, deep cerebellar nuclei; ECu, external cuneate nucleus; iEGL, inner EGL; IGL, inner GC layer; IO, inferior olivary nucleus; ns, nigrostriatal tract; oEGL, outer EGL; PCL, PC layer; Pn, pontine nucleus; pWM, presumptive white matter; py, pyramidal tract; scp, superior cerebellar peduncle; tz, trapezoid body; vsc, ventral spinocerebellar tract.

Perinatal (E17.5–P3) CB1 immunostaining is most prominent in long thin fibers cruising through the brainstem and the cerebellum, suggesting that the majority of CB1 localizes to elongating long-range axons at those developmental stages (Fig. 1*A–A’’*, green arrowheads). CB1-positive axon tracts within the brainstem at E17.5 are indicated by green shapes in summary diagrams (Fig. 1*C–C’’*). A dense mesh of CB1-positive fibers, consistent in appearance and distribution with pontocerebellar axons, can be seen within the presumptive white matter (pWM) in the developing cerebellar cortex at E17.5 (Fig. 1*A–A’’*). Zoomed-in views of CB1 immunolocalization within the cerebellum at this stage are shown in Figure 2, highlighting the distribution of CB1-positive fibers within cerebellar layers (Fig. 2*A*, *A’*).

Pontocerebellar afferents belong to two functionally and morphologically distinct categories: climbing fibers (CFs) and mossy fibers (MFs).

#### Climbing Fibers

CFs originate in the inferior olivary (IO) nuclei, located in the medial ventro caudal position in the brainstem. CB1 mRNA is robustly expressed within the IO (Fig. 1*B*, *C*, maroon), and fibers surrounding IO lamellae are labeled with CB1 (Fig. 1*A*, green arrowhead in IO). Zoomed-in view of CB1-positive fibers within IO is shown in Figure 2*C*. Based on the position and appearance of those fibers, they could be either neurites of IO projection neurons, or axons of IO afferents. However, no CB1 labeling is evident within the inferior cerebellar peduncles (icp; green dotted outline in Fig. 1*A’’*) through which axons of IO projection neurons enter the cerebellum. Thus, we conclude that at E17.5 IO neurons express CB1 mRNA, but CB1 protein is not expressed in the shafts of CF axons, and thus CB1 immunostaining in the cerebellar pWM is likely to represent primarily MFs, and not CFs. Possibly at this stage CB1 protein localizes to the developing terminals of CFs rather than axon shafts. In order to investigate this possibility, a detailed high-resolution analysis of the developing CF terminals could be performed in the future. It is also possible that CFs, which develop a few days ahead of MFs, have had CB1 expression in axon shafts at earlier developmental stages.

#### Mossy Fibers

MFs originate in numerous nuclei in the hindbrain and the spinal cord. Proprioceptive spinocerebellar afferents from the legs and trunk travel through the ventral pathway and enter the cerebellum through the superior cerebellar peduncle (scp; Fig. 1*A’*, green arrowhead), which is robustly labeled with CB1. Precerebellar inputs originating in the motor and association regions of the cerebral cortex are conveyed to the cerebellum via corticopontine inputs to the pontine nucleus (Pn; at the rostro-ventral surface of the brainstem). MFs originating in Pn run along the ventral and lateral surfaces of the brainstem and enter the cerebellum through the medial cerebellar peduncle (mcp; Fig. 1*A’*, *A’’*, green arrowheads), also robustly labeled with CB1. In addition, CB1 mRNA is strongly expressed in Pn (Fig. 1*B*, *B’*). Zoomed-in view highlighting radially oriented CB1-positive fibers within the Pn is shown in Figure 2*B*. Based on the evidence presented above, it is likely that the fibers labeled with CB1 in the pWM at E17.5 include a diverse population of developing MFs from multiple origins.

#### Additional structures prominently labeled with CB1 in the hindbrain at this stage

Cuneate (cu) and gracile (gr) fasciculi, which contain direct spinocerebellar MFs, are labeled with CB1 (Figs. 1*A*, *A’*, *C*, *C’*, green, 2*C*). Other exteroceptive and proprioceptive inputs from the upper and lower body are conveyed to the cerebellum through relay nuclei in the dorsal brainstem (Cu and Gr), which show CB1 mRNA expression (Fig. 1*B*, *B’*, *C*, *C’*, maroon). Interoceptive and ascending arousal inputs are relayed to the cerebellum through MFs originating in diverse nuclei of the reticular formation (RtTg, Rt, GiV, LRt, IRt, etc.), many of which express CB1 mRNA (Fig. 1*B’*, *B’’*, *C’*, *C’’*). Prominent CB1 labeling is seen in the pyramidal tracts (py) in the brainstem containing large ascending and descending axons connecting the cerebral cortex, brainstem and spinal cord, including the corticopontine fibers. The vestibulocerebellar nuclei, a source of vestibulocerebellar MFs, also prominently express CB1 mRNA (Fig. 1*B’’*, *C’’*, maroon).

#### Additional hindbrain nuclei prominently expressing CB1

In addition, robust immunoreactivity for CB1 is seen in the external layer of the dorsal cochlear nucleus (DC; Fig. 1*A’*, *A’’*, *C’*, *C’’*), a “cerebellum-like” cortical structure involved in the coordination of auditory versus non-auditory inputs. CB1 mRNA is prominently expressed in the dorsal nucleus of vagus nerve (X in Fig. 1*B’’*).

#### Differentiating granule cells

CB1 *in situ* hybridization at E18 (Fig. 1*B–B’’*) shows robust CB1 mRNA expression in small cells in the cerebellar cortex located below the PCL and above the pWM. Their tight packing and laminar localization suggest that they are IGL GCs. Thus, thinner and lighter stained CB1-positive fibers in the cerebellar cortex above pWM are likely the neurites of differentiating GCs (Fig. 2*A*, *A’*). Interestingly, this lighter CB1 expression in thin fibers is seen throughout the inner layers of the developing cerebellar cortex [inner EGL (iEGL), PCL, IGL], but not in the proliferative zone for GCs, the outer EGL (oEGL), suggesting that CB1 is expressed by differentiating, but not proliferating GCs. Dynamic and spatially restricted CB1 expression in the differentiating GCs during early postnatal developmental time course will be additionally discussed later.

### Abnormal distribution of axons in cerebellar peduncles and the developing cerebellar white matter in CB1 KOs

MFs navigate to the cerebellum through cerebellar peduncles, initially forming tight axonal fascicles deep within the cortex in the pWM, then defasciculating and spreading through more superficial layers, IGL and PCL, and stopping at the EGL boundary (Mason and Gregory, 1984; Ashwell and Zhang, 1998). Dark CB1 labeling in thicker fibers within cerebellar cortex, extending from pWM through IGL to PCL, but not to the EGL (Fig. 2*A*, *A’*), is consistent with morphologic descriptions of MFs. By P0, all MFs have already arrived in the cerebellar cortex, but their coverage continues to increase due to defasciculation and branching (Mason, 1985; Ashwell and Zhang, 1992). In the forebrain, CB1 KO mice exhibit defects in axonal fasciculation (Mulder et al., 2008; Wu et al., 2010). Since we observed robust expression of CB1 in axon shafts of cerebellar MFs, we asked whether, similarly to the phenotype described in the forebrain, cannabinoid signaling through CB1 receptors is required for regulation of MF fasciculation or spreading in the developing cerebellar cortex. In order to address this question, we performed a qualitative analysis of axon distribution in CB1 KOs.

**Figure 3.**
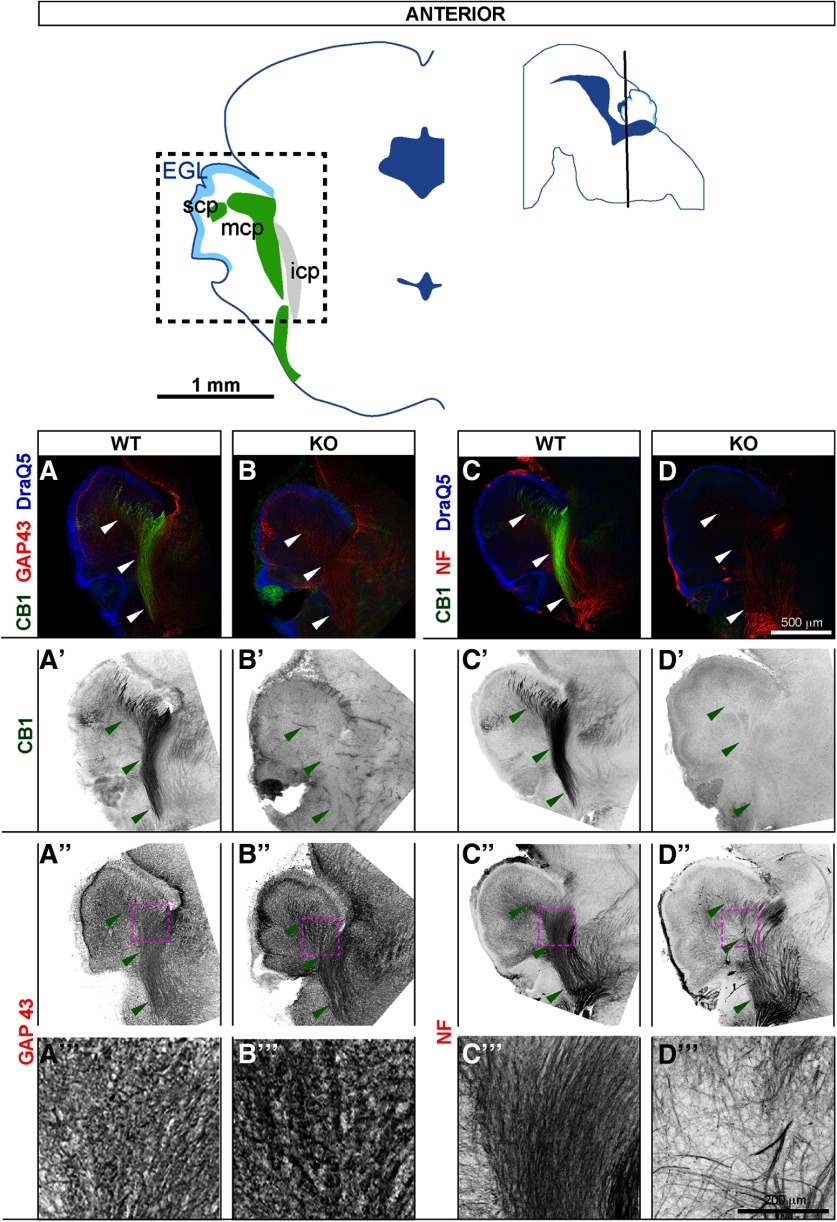
At E17.5, CB1 is highly expressed in cerebellar peduncles and is required for orderly arrangement of axons within the peduncles. Coronal sections at E17.5 through anterior cerebellar zone, scp and mcp are clearly seen in this plane of sectioning. Top row, left: outlines of a representative coronal section showing anatomic landmarks. Dotted square indicates the regions shown in panels ***A–D’’’***. Top row, right: black line through sagittal diagram shows plane of sectioning. Anatomical locations are adapted from Paxinos et al. (2007). ***A–A’’’***, In WTs, CB1 co-localizes with GAP43, a marker of elongating axons, and with axonal neurofilaments (***C–C”***). Large fascicles of CB1-positive axons run through scp and mcp (highlighted by arrowheads). ***B–B’’’***, ***D–D’’’***, CB1 staining is absent in KO littermates. Furthermore, density of GAP43 staining within the peduncles is increased in CB1 KOs (***A”***, ***A’’’*** vs ***B”***, ***B’’’***), and distribution of axons as judged by neurofilament staining appears abnormal (***C”***, ***C’’’*** vs ***D”***, ***D’’’***). Panels in the bottom row (***A’’’***, ***B’’’***, ***C’’’***, ***D’’’***) show zoomed-in views from regions indicated by maroon dotted outlines in (***A”***, ***B”***, ***C”***, ***D”***).

**Figure 4.**
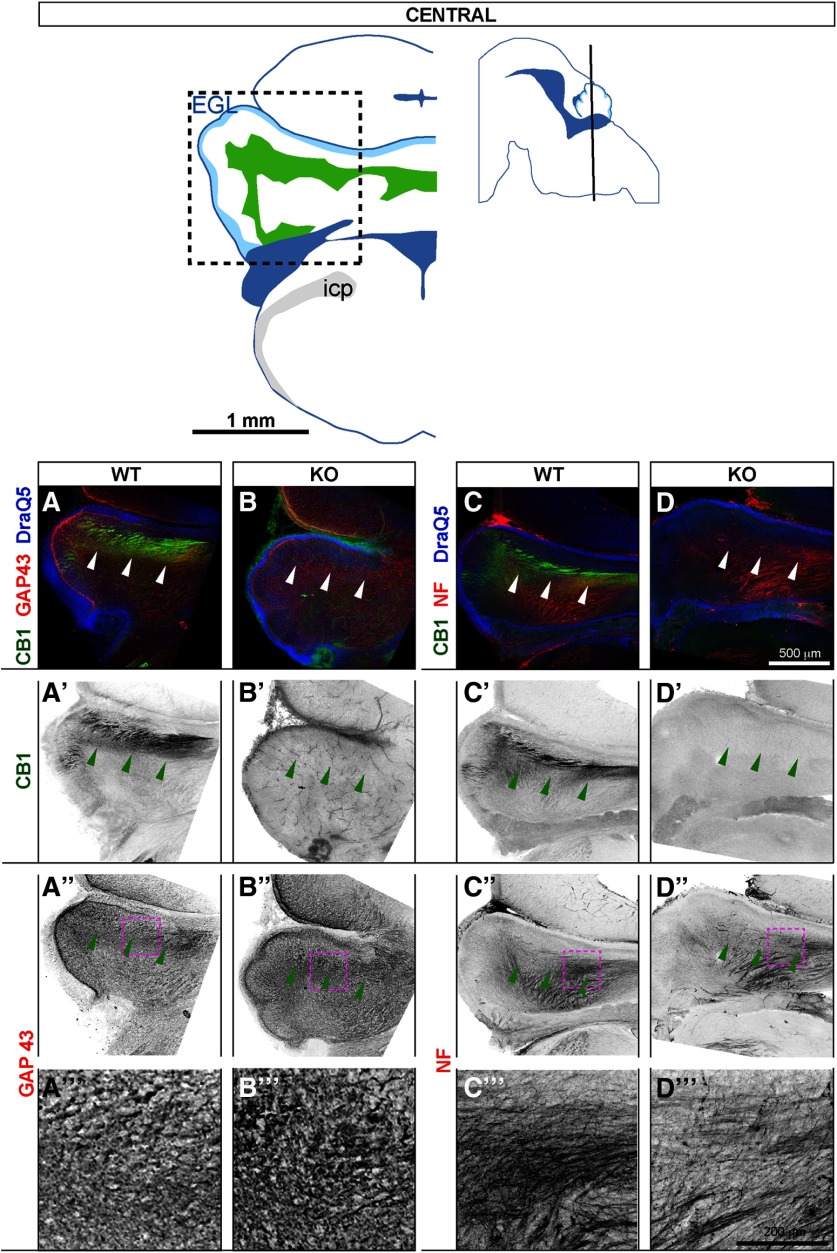
At E17.5, CB1 is expressed in the developing white matter tracts (pWM), which show increased GAP43 accumulation and altered axon distribution in CB1 KOs. Coronal sections at E17.5 through central cerebellar zone. Top row, left: outlines of a representative coronal section showing anatomic landmarks. Regions imaged in (***A–D’’’***) are indicated by dotted square. Top row, right: black line through sagittal diagram shows plane of sectioning. Anatomical locations are adapted from Paxinos et al. (2007). In WTs, CB1 co-localizes with axonal markers GAP43 (***A–A’’’***) and NF (***C–C’’’***). In KO littermates (***B–B’’’***) and (***D–D’’’***) CB1 staining is absent, density of GAP43 staining within pWM is increased (***B”***, ***B’’’***), and distribution of NF-positive axons (***D”***, ***D’’’***) is abnormal. Panels in the bottom row (***A’’’***–***D’’’***) show zoomed-in views from regions indicated by maroon dotted outlines in ***A”***–***D”***.

Serial coronal sections were collected at E17.5 from four KO and six WT E17.5 embryos from three litters originating from heterozygote to heterozygote crosses. Matching sections from anterior (Fig. 3, diagram showing plane of sectioning and anatomic landmarks is shown in top row) and central (Fig. 4, diagram showing plane of sectioning and anatomic landmarks is shown in top row) levels were co-stained with CB1 and two markers of elongating axons (GAP43, expressed in axon shafts and growth cones of actively growing axons), and pan-phosphorylated neurofilament antibody SMI312 (NF, enriched in long-range axons at all developmental stages).

Consistent with results shown in Figures 1, 2, in WT animals, CB1 is robustly expressed in mcp and scp (Fig. 3*A*, *A’*,*C*, *C’*), where it co-localizes with GAP43 (Fig. 3*A*, *A’’*) and NF (Fig. 3*C*, *C’’*). Confirming antibody specificity, CB1 staining is absent in KOs (Fig. 3*B*, *B’*, *D*, *D’*). GAP43 is broadly expressed in cerebellar peduncles and all cerebellar layers except EGL in all animals (Fig. 3*A–A’’’*, *B–B’’’*). Qualitative examination (six WT, four KO animals) reveals an increase in the intensity of GAP43 staining in CB1 KOs, suggesting either increased GAP43 expression, or increase in the number or density of GAP43 positive fibers. Zoomed-in views of GAP43 staining within cerebellar peduncles are shown in Figure 3*A’’’* (WT) and Figure 3*B’’’* (KO). Similar to CB1, NF staining is highly enriched in cerebellar peduncles, where NF-positive axons are evenly and broadly distributed in WTs (Fig. 3*C–C’’’*). In CB1 KOs, NF-positive axons are unevenly distributed within cerebellar peduncles, with dense NF-positive clusters intervening with NF-negative patches (Fig. 3*D–D’’’*). Zoomed-in views of NF staining within cerebellar peduncles are shown in Figure 3*C’’’*, *D’’’*. Similarly, sections through the central zone (Fig. 4) show increased density of GAP43 staining in pWM in CB1 KOs as compared with littermate WTs (Fig. 4*A’’*, zoomed-in view in Fig. 4*A’’’*, Fig. 4*B’’*, zoomed-in view in Fig. 4*B’’’*); as well as abnormal distribution of NF-positive axons (Fig. 4*C’’*, zoomed-in view in Fig. 4*C’’’*, Fig. 4*D’’*, zoomed-in view in Fig. 4*D’’’*). Thus, using two distinct markers of elongating long-range axons, we show that their expression and distribution are abnormal within cerebellar peduncles and the developing cerebellar cortex in CB1 KOs.

### During the first two postnatal weeks CB1 is expressed primarily in differentiating GCs

#### CB1 is expressed in processes and somata of radially migrating GCs

**Figure 5.**
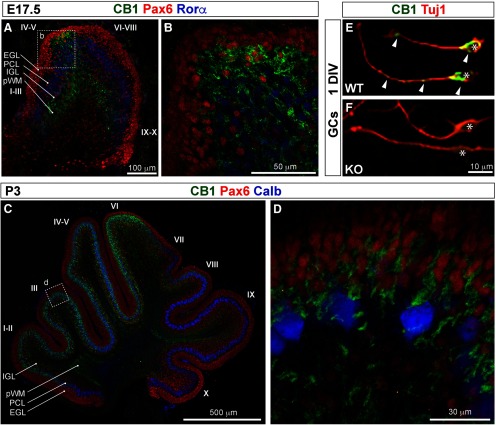
CB1 is highly expressed in a subset of differentiating GCs during the first postnatal week. Midsagittal sections of cerebellar vermis at E17.5 (***A***, ***B***) and at P3 (***C***, ***D***). ***B***, ***D***, Zoomed-in views from regions highlighted by white dotted outlines in ***A***, ***C***. During the first postnatal week, CB1 expression (green) becomes pronounced in differentiating GCs in the anterior and central cerebellar vermis. Postmitotic GCs in the EGL and GCs radially migrating through the PCL are marked by expression of transcription factor Pax6 (red in ***A*–*D***). PCs (PCL) are identified by expression of Rorα at E17.5 (blue in ***A***, ***B***) and calbindin (Calb) expression at P3 (blue in ***C***, ***D***). ***A***, At E17.5, CB1 expression is most prominent in a patch of radially migrating GCs clustered at the tip of developing lobes IV and V. ***C***, At P3 the territory of radially migrating GCs that express high levels of CB1 expands to occupy the extent of lobes I–VI, but not the posterior and nodular zones (lobes VII–X). Higher magnification images (***B***, ***D***) highlight CB1 expression in neurites surrounding Pax6-positive nuclei at the inner boundary of EGL and throughout the PCL and the developing IGL. ***E***, CB1 is expressed in neurites and somata of isolated and purified 24-h-cultured (1 DIV) GCs. GC neurites and somata are identified by expression of neuronal tubulin (Tuj1, red). Somata of GCs are indicated by asterisks. Arrowheads highlight CB1 expression in somata, axons, and growth cone of 1 DIV GCs. ***F***, Confirming specificity of CB1 expression in differentiating GCs, the staining is absent in sister GC cultures isolated from CB1 KOs.

At E17.5 CB1 immunohistochemistry reveals patches of radially oriented fibers extending from iEGL through the PCL to the IGL in the central zone corresponding to lobes IV–VI (Fig. 5*A*; zoomed-in view of the tip of lobes IV–V is shown in Fig. 5*B*). Restricted CB1 localization in radially oriented patches in iEGL/PCL/IGL at E17–P0 is reminiscent of GC “rashes” (Arndt et al., 1998; Redies et al., 2002) thought to distinguish clones of radially migrating GCs. Starting from P3, CB1 expression in thin fibers extending from the iEGL through the PCL to the IGL is prominent throughout the anterior and central zones (lobes I–VI, Fig. 5*C*; a zoomed-in view of the tip of lobe III is shown in Fig. 5*D*). Morphological characteristics of CB1-positive fibers, and their distribution relative to cerebellar layers, suggest that CB1 is expressed in radially migrating and differentiating GCs in the anterior and central (lobes I–VI) cerebellar zones, but not the posterior and nodular (lobes VIII–X). Interestingly, CB1 expression is seen in the iEGL, but not the oEGL, suggesting that high levels of CB1 are expressed by differentiating, but not proliferating GCs. Indeed, double-labeling with a marker of postmitotic GCs, Pax6 (Fig. 5*A–D*, red), shows CB1-positive fibers (Fig. 5*B*, *D*, green) surrounding Pax6-positive nuclei of GCs in the iEGL and the PCL. To confirm that CB1 localizes to neurites of differentiating GCs we stained GCs cultured *in vitro* for 24 h (1 DIV). The protocol that we use for isolation of GCs produces >90% pure and developmental stage synchronized GC culture (Manzini et al., 2006; Lee et al., 2009). As soon as isolated GC progenitors attach to coverslips they begin differentiating, thus a 1 DIV culture corresponds to ∼24-h-old postmitotic GCs. CB1 staining (Fig. 5*E*, arrowheads) is seen in somata (Fig. 5*E*, *F*, asterisks), extending neurites and in growth cones of 1 DIV GCs from WT (Fig. 5*E*), but not CB1 KO cultures (Fig. 5*F*).

#### CB1 expression in GCs during the first two postnatal weeks is largely restricted to anterior and central vermis and paravermis

**Figure 6.**
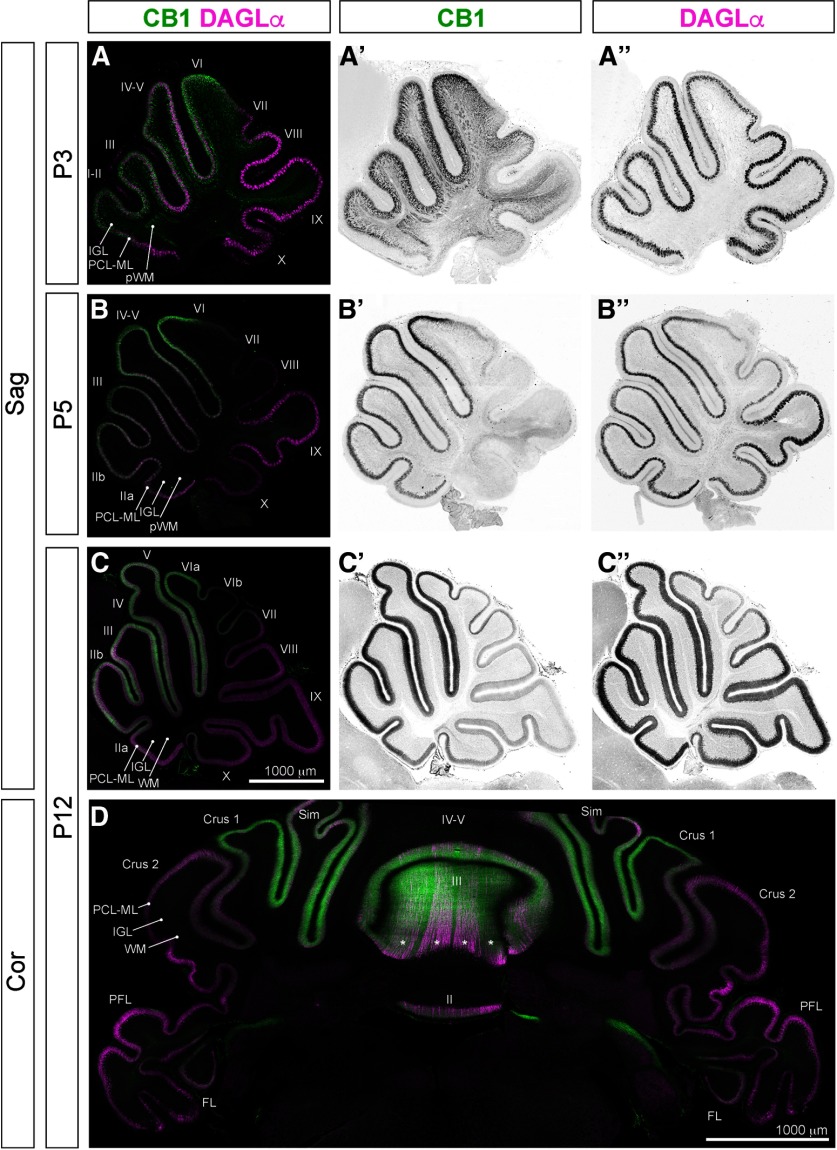
CB1 expression in developing cerebellum is dynamic and region specific, while DAGLα is expressed primarily in the PCL throughout development. CB1 expression in long-range axons within pWM becomes weak/undetectable after P5 (***A’*** compared to ***B’***, ***C’***). At P3 CB1 staining can be detected in PCL-IGL, corresponding to expression in migrating and differentiating GCs (***A***, ***A’***); however, at P5 (***B***, ***B’***) and P12 (***C***, ***C’***, ***D***), CB1 expression becomes restricted to ML in the anterior and central vermis. DAGLα (maroon) is expressed in PCL throughout cerebellar cortex (grayscale DAGLα channel in ***A”***, ***B”***, ***C”***). ***D***, In coronal sections, at P12, CB1 expression in ML is prominent in the vermis and paravermis (lobes II, III, IV–V, and Sim), but weak in the hemispheres (Crus 2) and in the nodular zone (PFL and FL). Stripes of high-DAGLα-expressing PCs are apparent in lobes III and IV–V (***D***, asterisks).

During the first two postnatal weeks CB1 shows a strong gradient of expression across different cerebellar regions: high-CB1-expressing GCs are concentrated in the anterior and central vermal zones, with low or no expression in the posterior and nodular zones and in the hemispheres (Fig. 6). Furthermore, CB1 expression in the developing cerebellum is very dynamic. Hence, at P3 CB1-expressing long-range axons can still be seen in the pWM (Fig. 6*A*, *A’*), but by P5 CB1 expression in pWM can no longer be detected (Fig. 6*B*, *B’*). As GCs mature, subcellular distribution of CB1 seems to shift from neurites and somata of radially migrating GCs at P3 (Fig. 6*A*, *A’*) to GC axons within the ML at P5 and P12 (Fig. 6*B*, *B’*, *C*, *C’*). CB1 expression in ML is prominent in vermal lobes I–VI and lobes Sim and Crus 1, but not in the posterior and nodular zones or the hemispheres (lobes VII–X, FL PFL, Crus 2) at P5-P12 (Fig. 6).

#### CB1 is expressed in PFs of differentiating GCs

**Figure 7.**
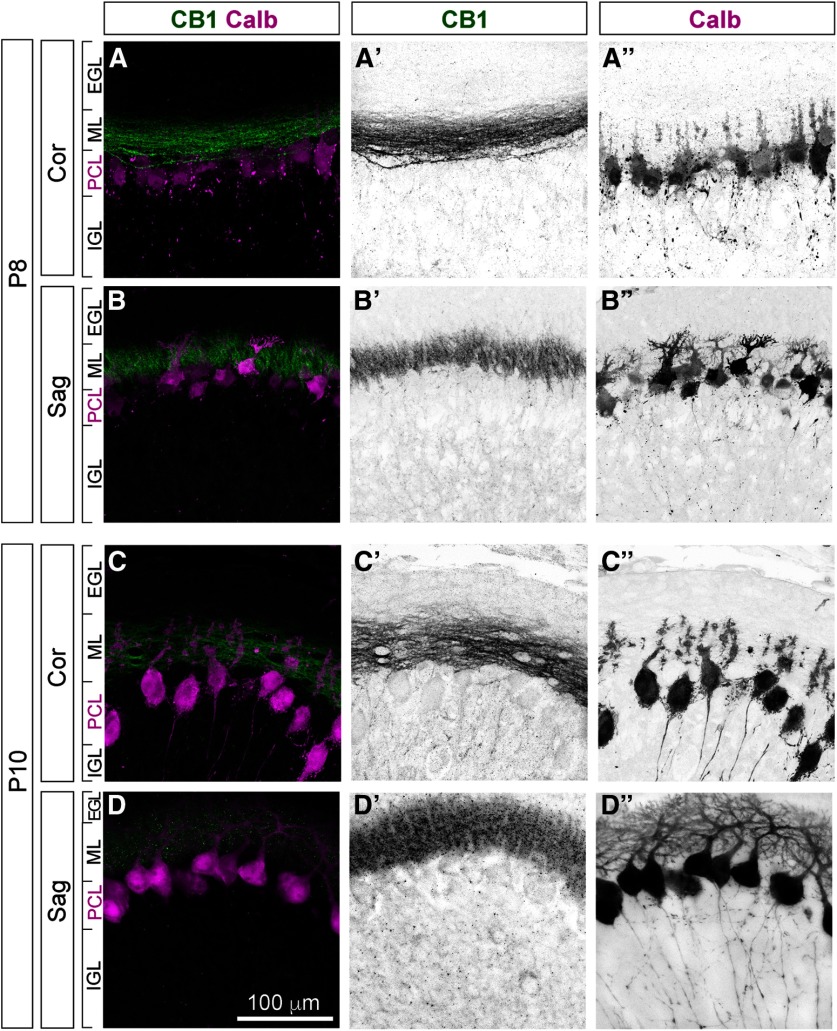
CB1 is expressed in developing PFs during the second postnatal week. High-magnification views of CB1 localization relative to cerebellar layers. Images were taken at the tips of lobe III. ***A–A”***, ***C–C”***, Coronal sections, in-plane with trajectories of PFs and orthogonal to the plane of branching of PC dendrites. ***B–B”***, ***D–D”***, Sagittal sections, orthogonal to the trajectories of PFs, and in-plane with branching of PC dendritic trees. During the second postnatal week (P8, top two rows; P10, bottom two rows) strong CB1 expression (green) is most prominent in ML, in thin fibers stacked above PCL (marked by Calb, maroon) running parallel to the coronal plane of sectioning, i.e., in the PFs, axons of GCs. No co-localization of CB1 is seen with PC dendrites, somata, or proximal axons. CB1 expression was not detected in EGL. Thin CB1-positive fibers and week somatic staining in the IGL (more prominent at P10; ***C’***, ***D’***) most likely correspond to weak expression in GC somata and in vertical portions of GC axons.

During the second postnatal week the most prominent expression of CB1 is seen within ML (Fig. 6*B–D*). ML contains dendrites of PCs and axons of GCs, the PFs, as well as somata, axons, and dendrites of differentiating ML interneurons. Higher magnification images were taken at the tip of lobe III in both coronal and sagittal planes to distinguish morphological characteristics of CB1-positive fibers (Fig. 7). CB1 (green) is expressed in thin straight fibers stacked above the PCL (marked by Calb; Fig. 7, maroon) running parallel to the coronal plane of sectioning, i.e., matching morphological characteristics of PFs.

### DAGLα, a major biosynthetic enzyme of 2-AG in the brain, is expressed in PCs at all developmental stages examined

**Figure 8.**
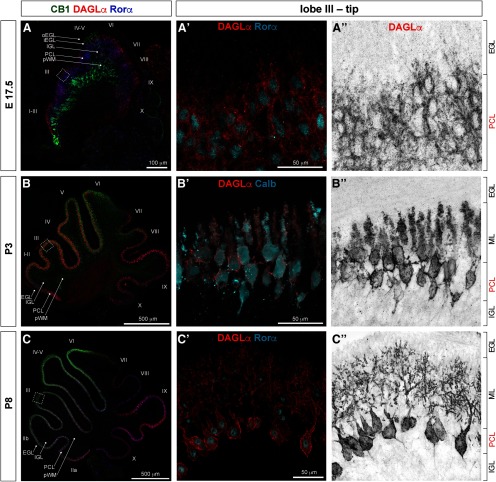
DAGLα is expressed in PCs during cerebellar development. Midsagittal sections of cerebellar vermis at (***A–A”***) E17.5, (***B–B”***) P3, and (***C–C”***) P8. Middle and right columns, Zoomed-in views at the tip of lobe III in the anterior zone. At all developmental stages investigated, immunoreactivity for DAGLα (red) within cerebellar cortex was restricted to somata, dendrites, and initial axon segments of PCs (identified by morphology and by expression of Rorα and Calb, blue/cyan).

DAGLα expression within the cerebellar cortex is largely confined to the PCL in all cerebellar regions and zones, and at all developmental stages investigated (Fig. 6*A*, *A’’*, *B*, *B’’*, *C*, *C’’*,*D*). Many molecules that localize to the PCL exhibit a “striped” pattern of expression characterized by interspersed anteroposterior stripes of positive and negative PCs (Apps et al., 2018). Similarly, DAGLα expression exhibits striping in the anterior zone at P12 (Fig. 6*D*). Distribution of DAGLα relative to PC outlines suggests that it localizes to plasma membranes (higher magnification images taken at the tips of lobe III [Fig. 8*A’*, *A’’*, *B’*, *B’’*, *C*, *C’’*; PCs are identified by expression of Rorα (Fig. 8*A–A’’*, *C–C’’*) and Calb (Fig. 8*B–B’’*), cyan]. DAGLα is highly expressed in somata, dendrites, and axon initial segments, but not in axon shafts or axon terminals of PCs.

### MAGLα is expressed in the PC somatodendritic compartment, and in PC axons

**Figure 9.**
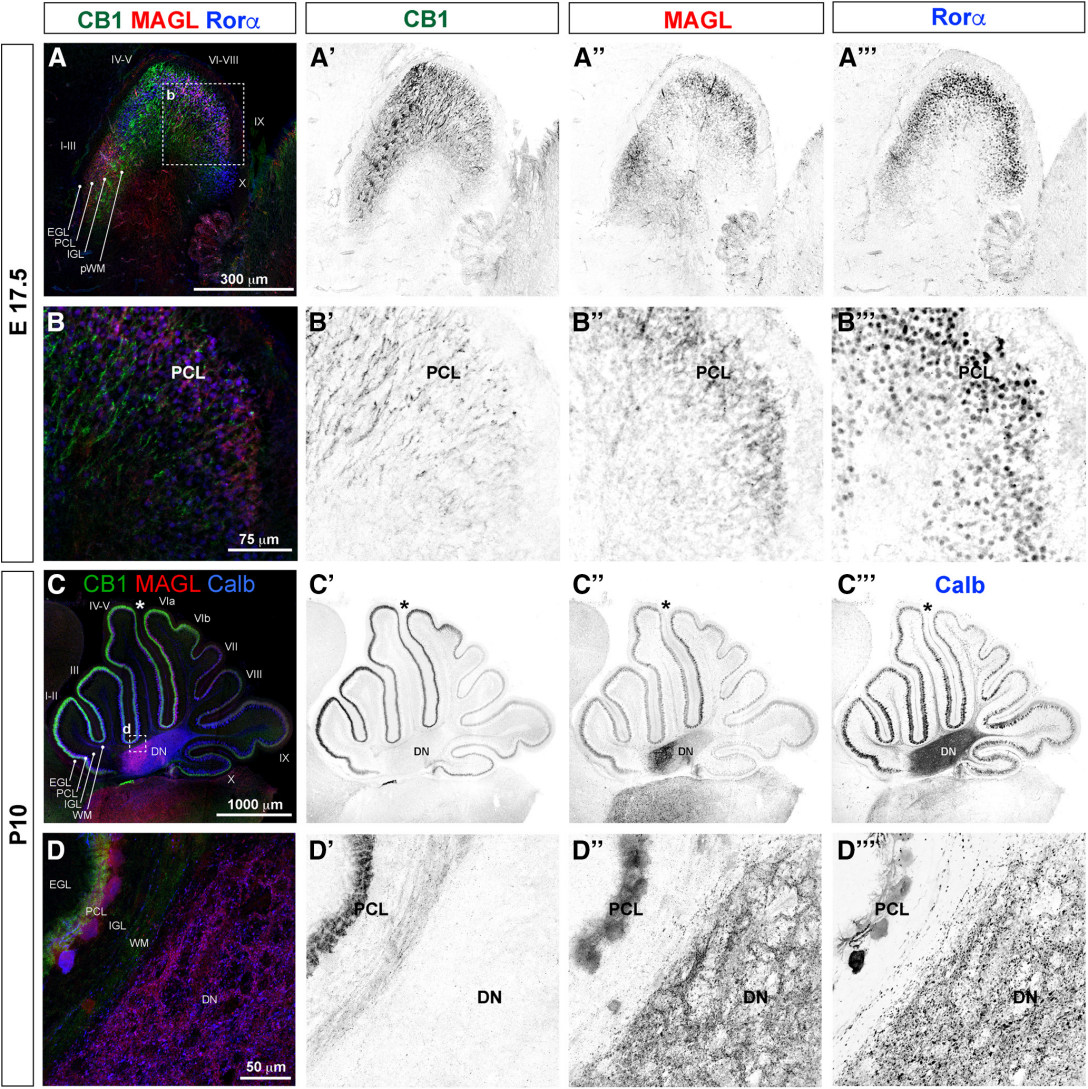
MAGL is expressed in PCs. Midsagittal vermal sections at (***A–A’’’***, ***B–B’’’***) E17.5 and (***C–C’’’***, ***D–D’’’***) P10. ***B–B’’’***, ***D–D’’’***, are zoomed-in views from regions indicated by white dotted lines in ***A***, ***C***. PCs are identified by Rorα (***A***, ***B***, blue) and Calb (***C***, ***D***, blue). MAGL (red) expression is seen primarily in the PCL (***A***, ***A”***, ***C***, ***C”***). Higher magnification views show that MAGL is expressed in the somata and dendrites of PCs (***B***, ***B”***, ***D***, ***D”***, PCL) and in axon terminals in DN (***C”***, ***D”***). MAGL expression at P10 is uneven across the midvermal region, with the highest levels expressed within PCs lining the primary fissure, which separates lobes V and VI (***C–C’’’***, asterisks).

The availability of 2-AG depends on the distribution of its synthesizing and degrading enzymes. Therefore, we went on to characterize the developmental expression of MAGL. MAGL expression in the developing cerebellum is seen primarily in the PCL at all stages investigated (Fig. 9*A–D*). Thus, a balance of DAGLα and MAGL expression and activity, regulated cell-autonomously in PCs, would determine the amount of 2-AG available to activate CB1 receptors. Interestingly, levels of MAGL expression differ between cerebellar zones. Particularly striking is the enrichment of MAGL at P10 in PCs lining the primary fissure (prm), which separates lobes V and VI (Fig. 9*C*, *C’’*, prm is indicated by asterisk), and is within the caudal boundary of CB1-positive GC territory (Fig. 9*C*, *C’*). Interestingly, MAGL expression is prominent in PC axon terminals in deep cerebellar nuclei (DN; Fig. 9*C–C’’’*; zoomed-in view in Fig. 9*D–D’’’*; PC axon terminal in DN are identified by Calb expression), while CB1 expression was not detected within DN at this developmental stage (P10; Fig. 9*C*, *C’*, *D*, *D’*).

### Genetic loss of CB1 leads to a reduction in the cerebellar vermis area

**Figure 10.**
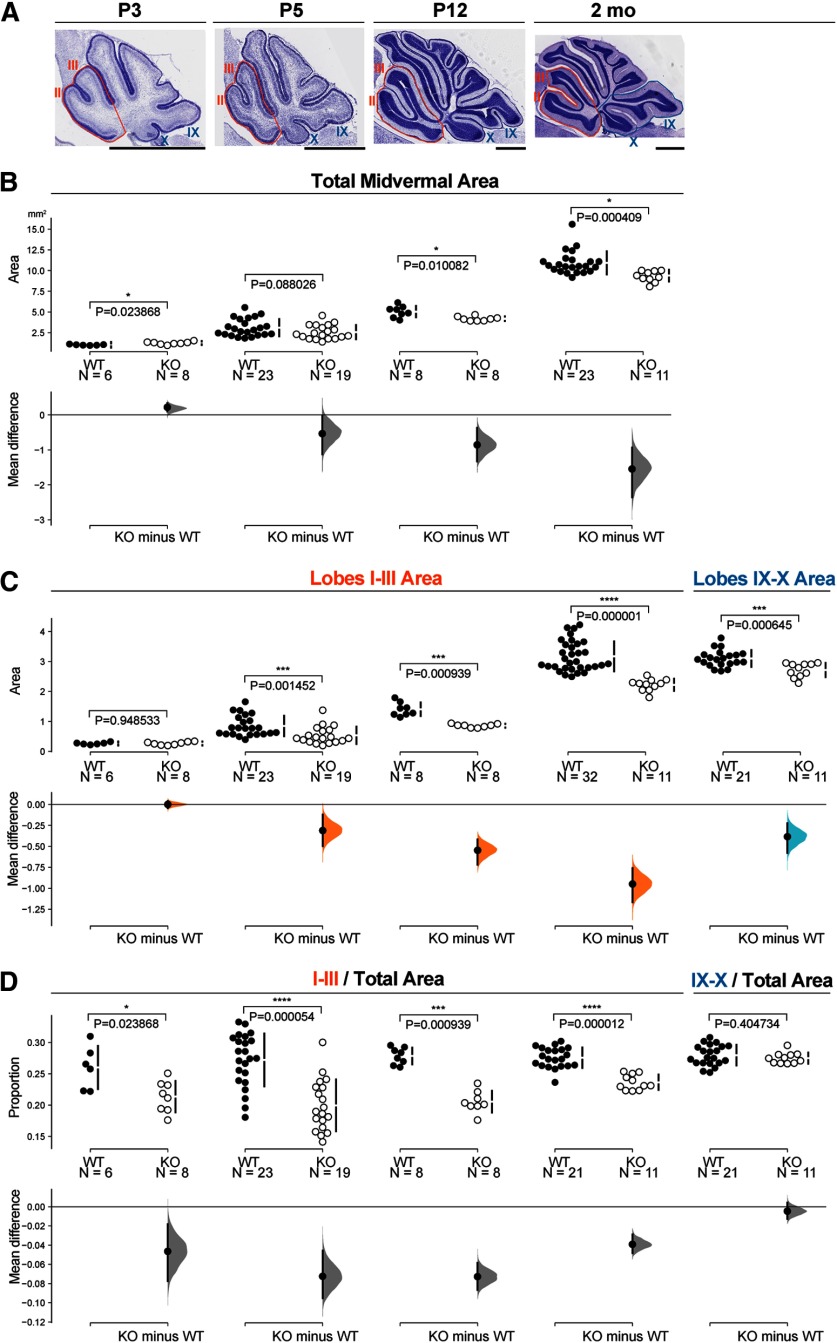
Midvermal area is reduced in CB1 KOs. ***A***, Representative Nissl-stained midvermal cerebellar sections from WTs, showing regions used for area quantification and illustrating postnatal developmental stages analyzed. Territories of anterior lobes I–III, and nodular lobes IX–X are outlined. Scale bars: 200 μm. ***B*–*D***, The mean difference for (***B***) four comparisons (P3, P5, P12, two months) and (***C***, ***D***) five comparisons (lobes I–III P3, lobes I–III P5, lobes I–III P12, lobes I–III two months, lobes IX–X two months) are shown in Cumming estimation plots. The raw data are plotted on the upper axes; each mean difference is plotted on the lower axes as a bootstrap sampling distribution. Mean differences are depicted as dots; 95% confidence intervals are indicated by the ends of the vertical error bars. A total of 5000 bootstrap samples were taken; the confidence interval is bias-corrected and accelerated. Details of statistical analysis are shown in Table 3. ***B***, Stunted increase in total midvermal area in CB1 KOs becomes more pronounced with age. ***C***, ***D***, Reduction in midvermal area in CB1 KOs is primarily contributed by reduced size of anterior zone, quantified for lobes I–III.

In order to assess whether eCB signaling is involved in the regulation of gross anatomical aspects of cerebellar development, we analyzed the areas of the cerebellar midvermis through a developmental time course (P3, P5, P12, and two-month-old) in CB1 KOs and WT littermates. Figure 10*A* shows Nissl-stained midvermal cerebellar sections from WTs at these developmental stages. Regions used for area quantification are highlighted (lobes I–III in the anterior zone, Fig. 10*A*, orange outline; lobes IX–X in the nodular zone, Fig. 10*A*, blue outline). Our initial analysis revealed no statistically significant differences and largely overlapping distribution of values between sexes (data not shown), hence we show sex-combined data in Figure 10.

The cerebellum undergoes a protracted postnatal development, and the majority of cerebellar GCs in mice are generated during the first two postnatal weeks from a progenitor pool that is seeded embryonically. Our results highlight about a 15% reduction in the midvermal area in two-month-old CB1 KOs (Fig. 10*B*, right column). Reduction in the midvermal area could be a consequence of smaller progenitor pool in embryonic cerebellar primordium, a reduced proliferation (or increased apoptosis) of progenitors, or an altered differentiation of postmitotic cerebellar neurons or glia. Since progenitor pools are seeded embryonically, we expected to see a reduction in the midvermal area in CB1 KOs at birth, if decreased proliferation or increased apoptosis of progenitors were contributing to reduced cerebellar growth. However, at P3 cerebellar midvermis is actually slightly larger in KOs as compared with WT littermates (Fig. 10*B*, left column). Thus, a reduction in the midvermal size that is observed in two-month-old CB1 KOs can be attributed to the differences that manifest during postnatal cerebellar development.

In WTs midvermal area expands ∼11-fold during postnatal development, from an average of 1 μm^2^ at P3 to ∼11 μm^2^ at two months (Fig. 10*B*, black dots). In CB1 KOs, postnatal expansion of the midvermal area is stunted with only ∼9-fold increase (Fig. 10*B*, white dots). Thus, the reduction in the total midvermal area in two-month-old KOs is a consequence of a reduced rate of postnatal vermal growth, which exhibits slower expansion after the first postnatal week in CB1 KOs (Fig. 10*B*).

Since CB1 expression is particularly prominent in GCs in the anterior-central cerebellar vermis, we assessed differences in the lobes I–III areas between KOs and WTs (Fig. 10*C*). CB1 KOs exhibit >30% area reduction of lobes I–III in two-month-olds (Fig. 10*C*). On the other hand, CB1 expression in differentiating GCs in the nodular zone (lobes IX–X) was very low/undetectable during the time course that we analyzed. Evaluating the difference in the mean areas of lobes IX–X in WTs and KOs we found a moderate decrease in size (Fig. 10*C*, last column), proportional with the reduction in the total midvermal areas (Fig. 10*D*, last column). In contrast, reduction in areas of lobes I–III is more dramatic, disproportionally contributing to the reduction in total cerebellar areas (Fig. 10*D*). The regional bias of the anatomic phenotype described here suggests that CB1 expression in differentiating GCs is important for the regulation of postnatal cerebellar growth.

### Cerebellar-influenced behaviors are selectively affected in CB1 KOs

**Figure 11.**
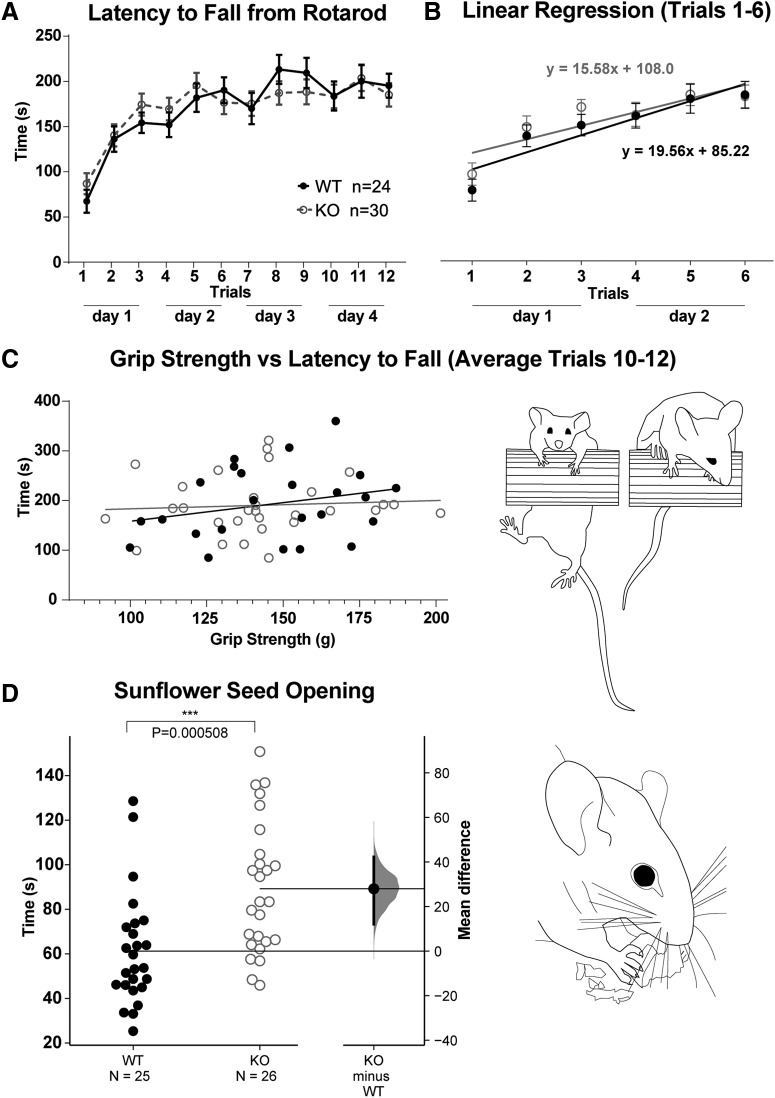
Selective impairments in motor behaviors in CB1 KOs at two-month-old. ***A***, Time course of rotarod performance (latency to fall from rotating rod accelerating from 4 to 40 rpm) was plotted per trial as average performance of all WT animals (24, sexes combined) and all KO animals (30, sexes combined). Three trials were performed per day over the course of 4 d (12 trials total). Both groups performed at comparable levels in the beginning of the time course (day 1, trials 1–3), in the subsequent trials (days 2 and 3, trials 4–9), and achieved similar performance in the fourth (last) day of the time course (trials 10–12). Statistical hypothesis of equivalent latency to fall between genotypes was shown to be true by Tukey multiple comparison, and by comparing areas under the curve for all trials (statistical table in Table 4). ***B***, Improvement in rotarod performance was evaluated by fitting linear regression curves over the first six trials; differences in slopes between genotypes were compared with evaluate rate of learning, and found to be similar between CB1 KOs and WTs. ***C***, No difference in grip strength was detected between genotypes, and performance in rotarod does not show grip-strength-dependent bias. ***D***, Mean differences in latency to open sunflower seeds between WTs (25 animals, sexes combined) and KOs (26 animals, sexes combined) are shown in Gardner–Altman estimation plot. Both groups are plotted on the left axes; the mean difference is plotted on floating axes on the right as a bootstrap sampling distribution. The mean difference is depicted as a dot; the 95% confidence interval is indicated by the ends of the vertical error bar. A total of 5000 bootstrap samples were taken; the confidence interval is bias-corrected and accelerated. Average time to open sunflower seeds is significantly longer in CB1 KOs. Details of statistical analysis are shown in Table 4.

Since cerebellar trauma and developmental malformations often lead to motor deficits, particularly when the anterior vermis is involved, we set out to characterize cerebellar-influenced motor behaviors in CB1 KO mice. Rotarod is considered a classical test of sensorimotor coordination, and a repeated rotarod challenge over a course of several days is routinely used as a measure of motor learning. The performance of two-month-old CB1 KO mice in the accelerating rotarod was indistinguishable from WT littermates (Fig. 11*A*), and CB1 KOs exhibited the same rate of learning in the rotarod task as WT littermates (Fig. 11*B*).

Notably, mild cerebellar damage in humans is associated with more pronounced deficits in fine-motor than gross-motor tasks (Manto, 2018). Thus, we also examined the performance of two-month-old CB1 KOs in the forelimb coordination sunflower seed opening task. In this experiment, mice that have been acclimated to sunflower seeds (to reduce effects of novelty anxiety) were timed from the moment they picked up a seed through time spent opening and consuming the seed. Only seeds that were mostly (>75%) consumed were included in the average time calculated for each mouse, eliminating possible confounding effects of differences in appetite, motivation or attention. There were no differences in the average number of seeds approached or consumed between genotypes (data not shown). CB1 KOs take longer to open seeds (Fig. 11*D*), indicating a significant deficit in forepaw coordination in CB1 KOs. We observed no differences in forelimb strength between genotypes, and no correlation between forelimb strength and rotarod performance (Fig. 11*C*).

## Discussion

Prior work characterizing localization of CB1 noted its prominent expression in the adult cerebellum (Herkenham et al., 1990, 1991b; Kawamura et al., 2006). In this study, we find that CB1 is also robustly expressed during cerebellar development. Using immunohistochemistry with KO-verified antibodies and molecular markers of cerebellar cell types, we show a developmentally dynamic, region and cell-type-specific pattern of CB1 expression in E17.5–P12 mouse cerebella. Immunolocalization of DAGLα and MAGL to PCs emphasizes the key role of PCs in the production of 2-AG eCB ligand. Anatomical analysis of CB1 KO and WT littermates from heterozygote crosses showed a reduction in anterior vermis size in CB1 KOs, which is more pronounced later in postnatal development and in adults. CB1 KOs exhibit impairments in sunflower seed opening (sensorimotor coordination task involving forepaws and face) influenced by anterior cerebellar vermis and paravermis.

### Perinatal localization of CB1 in developing long-range precerebellar axons and their disorganized distribution in CB1 KOs suggest a widespread role of eCB signaling in axon development

Our results show that at late embryonic and early postnatal stages (E17.5–P3) CB1 is prominently expressed in long-range axons in the brainstem and in the cerebellum. This is consistent with autoradiographic studies performed in rat and human embryos showing hindbrain localization of CB1 primarily in long-range axon tracts (Berrendero et al., 1998; Mato et al., 2003). We expand those early observations to the perinatal period in mice and provide a high-resolution cellular characterization of CB1 distribution in pontocerebellar axons. Furthermore, we show qualitative analysis of GAP43 and NF-expressing axons in the cerebella of CB1 KO mice as compared with WT littermates at birth, revealing higher expression of GAP43 in KOs, possibly reflecting the earlier developmental stage of the axons; and defects in axon distribution within the cerebellar peduncles and in the pWM in CB1 KOs. However, the magnitude of the effect cannot be estimated without quantitative analysis, and given inherent variability between individuals, it is very important to repeat this analysis in the future with a larger sample. Keeping those limitations in mind, we cautiously propose that signaling through CB1 regulates fasciculation of pontocerebellar axons, similarly to the previously described role of eCB signaling in the regulation of thalamocortical and subcortical axon fasciculation (Mulder et al., 2008; Wu et al., 2010). Thus, high expression of CB1 in elongating long-range axons, and its requirement for regulation of axon fasciculation, appears to be a widespread property of the developing mammalian nervous system, prominent both in the forebrain and the hindbrain.

MFs spread postnatally throughout the developing cerebellar cortex due to defasciculation and branching, expanding through the PCL, and stopping at the EGL boundary (Mason, 1985; Ashwell and Zhang, 1992). Their targeting specificity within subregions, layers, and cell types is being refined throughout the first three postnatal weeks (Kalinovsky et al., 2011; Kano et al., 2018). Thus, early defects in defasciculation identified in this work could affect synaptic targeting and regional specificity later in development, thus affecting computational properties of microcircuits within the cerebellar cortex and the shape of WM tracts in the adult.

We show that in the perinatal cerebellum DAGLα is continuously expressed by PCs, thus PCL is likely to be a source of high concentrations of 2-AG available to activate CB1 receptors in neurites approaching or traversing the PCL. Interestingly, in the forebrain, two modes of eCB signaling have been described in the regulation of neurodevelopment: autocrine/cell-autonomous and paracrine. Functional dichotomy between the two modes has been proposed in the developing forebrain: (1) autocrine mode is characteristic of earlier developmental decisions (proliferation, apoptosis, axon growth and fasciculation; Aguado et al., 2005; Mulder et al., 2008; Watson et al., 2008); (2) while retrograde-target-derived mode becomes predominant at later developmental stages and contributes to growth cone guidance and synaptic maturation (Berghuis et al., 2007). Contrary to observations in the forebrain, our immunohistochemical analysis revealed no expression of DAGLα in CB1-positive axons in the cerebellum or in the brainstem, thus our current observations are more consistent with paracrine signaling model. However, comprehensive characterization of the sources of eCB signaling at this developmental stage was beyond the scope of this work. Future studies should address whether additional 2-AG synthetizing enzymes, such as not DAGLβ or enzymes synthetizing anandamide, another classical eCB, are expressed in the developing cerebellum.

### High expression of CB1 in developing GCs is restricted to the anterior vermis, identifying a molecularly distinct subpopulation of GCs

By P5 CB1 expression becomes very low in the long-range axons in pWM, but very prominent in neurites of radially migrating GCs and in the extending and maturing PFs in the anterior and central zones. Our study reveals that during the first two postnatal weeks differentiating GCs expressing high levels of CB1 are prominent in the anterior and central cerebellar vermis and paravermis, but not in the other cerebellar zones. Early autoradiographic localization studies in cerebellar mutants characterized by primary degeneration of PC versus GCs highlighted robust expression of CB1 in GCs (Herkenham et al., 1991a). Subsequent functional studies in GC-specific conditional CB1 mutants (Carey et al., 2011) underscored the physiological significance of CB1 expression in the PFs of GCs in the adult. A key role of retrograde eCB signaling from PCs to PFs has been implicated in both cerebellar LTD (Safo and Regehr, 2005) and DSE (Kreitzer and Regehr, 2001; Tanimura et al., 2009). However, spike timing parameters required for PF LTD vary significantly between the vermis and other cerebellar regions (Suvrathan et al., 2016), leading to a proposal that heterogenous (cannabinoid dependent and independent) mechanisms may contribute to PF plasticity in the vermal versus non-vermal cerebellar regions (Crepel, 2007; Suvrathan and Raymond, 2018). Our results, highlighting a spatially restricted pattern of CB1 expression in developing PFs during the first two postnatal weeks, offer molecular basis in support of the hypothesis that PF plasticity in hemispheres and nodular zones may be less dependent on eCB signaling than in the anterior and central vermis.

Boundaries of molecularly distinct PC subpopulations coincide with borders of functional subregions (Sillitoe et al., 2010). However, molecular and functional subpopulations of GCs in the IGL are only beginning to be identified. Our results show that high CB1 expression defines the anterior-central vermal population of GCs during the first two postnatal weeks, identifying a novel molecular marker that could aid in future research into specific mechanisms distinguishing functional cerebellar subregions within the IGL. Enrichment in MAGL expression in PCs lining the primary fissure is likely to cause higher rates of 2-AG hydrolysis in the adjacent tissue, dampening eCB signaling through CB1 in differentiating GCs within lobes V–VI, and potentially creating a sharp boundary of eCB signaling effects, confining them to the anterior-central zones.

### Are other components of the cannabinoid signaling system expressed during cerebellar development?

The cannabinoid system includes multiple G-protein coupled receptors and ion channels, protein kinase signaling pathways, and a host of synthesizing and degrading enzymes, whose localization and function have not yet been comprehensively characterized during cerebellar development. In this study, we conducted an initial characterization of the key components of ECS (CB1, DAGLα, MAGL) involved in signaling mediated by 2-AG, the most abundant classical eCB, showing that they are expressed, and that cannabinoid signaling through CB1 receptor is required, for normal cerebellar development. Thus, this study lays the foundation for more comprehensive future characterization of expression and developmental roles of additional ECS components. For instance, although MAGL is considered to be the main degradation enzyme for 2-AG in the central nervous system, serine hydrolases ABHD6 and ABHD12, which are found in brain tissue, are also capable of degrading eCBs (Blankman et al., 2007; Marrs et al., 2010). Thus, it would be valuable in the future to characterize whether ABHD hydrolases are expressed, and contribute to the regulation of 2-AG availability, in the developing cerebellum.

The abundance of the other classical eCB, anandamide, is regulated by multiple, potentially redundant, anabolic and catabolic pathways (Maccarrone, 2017). An interesting molecular target for initial characterization of the expression and function of anandamide in cerebellar development is *N*-acyl phosphatidylethanolamine-specific phospholipase D (NAPE-PLD), which was shown to play a role in the regulation of cerebellar anandamide levels (Leishman et al., 2016).

In addition, this work provides information on the developmental periods, cerebellar zones, and cell types expressing CB1, DAGLα, and MAGL, essential for the design of cerebellum-specific conditional KOs of these ECS components to address their cerebellar-specific contributions to brain development and behavioral regulation.

### Reduction in anterior cerebellar vermis size in CB1 KOs can be detected starting at the end of the first postnatal week, and persists into adulthood, suggesting that eCB signaling regulates vermal growth

The importance of eCB signaling in cerebellar development is highlighted by a significant gross anatomic phenotype apparent in CB1 KOs, demonstrating that eCB signaling regulates postnatal growth of cerebellar vermis. Reinforcing our observations of the regional specificity of high CB1 expression in differentiating GCs, the most pronounced effect is seen in the anterior vermis of CB1 KOs. The reduced size of the anterior cerebellar vermis may lead to profound functional consequences by affecting relative sizes of functional cerebellar subregions, the configuration of local microcircuits, or global patterns of long-range connectivity.

### How do alterations in vermal size affect behavior?

Motor dysmetria and ataxia are characteristic of neurodevelopmental conditions that involve vermal agenesis, hypoplasia or degeneration (for review, see Basson and Wingate, 2013). Characteristically, patients with cerebellar lesions exhibit greater deficits in motor behaviors that require more fine-motor control, or adjustments of timing or direction (Manto, 2018).

Since the anatomic analysis of CB1 KOs indicated the anterior vermis as the region where the KO phenotype is most pronounced, we chose to focus on motor tasks for the initial behavioral characterization. Our results show impairment in fine motor forepaw coordination (opening sunflower seeds) in CB1 KOs, yet no defects in the gross motor coordination task (accelerating rotarod). Our results are in agreement with previous studies of rotarod performance in CB1 KOs, which also found no impairments in young-adult mice (Steiner et al., 1999; Kishimoto and Kano, 2006). However, we expand previous characterization of cerebellar-influenced behaviors to include an additional sensorimotor coordination task, sunflower seed opening, which we show to be sensitive to alterations in eCB signaling. More pronounced deficits being observed in fine-motor rather than gross-motor task is consistent with mild cerebellar ataxia.

The nature of behavioral changes in CB1 KOs in conjunction with the vermal anatomic phenotype suggest that cannabinoid signaling plays a key role in cerebellar development. However, brain regions do not develop and function in isolation. The cerebellum receives afferents (mostly relayed through brainstem nuclei) from the spinal cord and cerebral cortex, which arrive in the cerebellar primordium before birth. Reciprocal signaling between afferent axons and developing cerebellar neurons can shape developmental trajectories in both the origin and the target brain regions. CB1 is broadly expressed and was shown to play important roles in forebrain development, including specification and axon growth of corticospinal neurons that convey corollary cortical inputs to the cerebellum through pontine nuclei (Díaz-Alonso et al., 2012). Since our study was performed in global *cb1*−/− mice, the phenotypes described here cannot be ascribed to cell-autonomous effects in the cerebellum.

Multiple reciprocal cortico-cerebellar and cerebello-cortical loops have been shown to control animal behavior, including forepaw coordination. For instance, subcortical projection neurons investigated in Díaz-Alonso et al. (2012) branch as they pass above pontine nuclei in the brainstem and make synapses with pontine neurons providing corollary inputs to the cerebellum through MFs. Conversely, cerebellar output controls the pattern of ongoing activity in subcortical projection neurons. In fact, with increasing task complexity, a higher degree of coordination of neural activity is observed between the cerebrum and cerebellum. Reciprocal connectivity between the cerebellum and cerebral cortex is established early during development and aberrant development of those long-range connections in CB1 KOs could contribute to the deficits in forepaw coordination observed in both (Díaz-Alonso et al., 2012) and our study. Thus, we propose that it is very likely that cannabinoid signaling in both regions is important for regulation of circuit development, and perturbations in the development of either of those regions can lead to abnormalities in similar behavioral measurements. Characterization of conditional cerebellar-specific KO of ECS components will help to resolve those questions in the future.

## Synthesis

Reviewing Editor: Lynda Erskine, University of Aberdeen Decisions are customarily a result of the Reviewing Editor and the peer reviewers coming together and discussing their recommendations until a consensus is reached. When revisions are invited, a fact-based synthesis statement explaining their decision and outlining what is needed to prepare a revision will be listed below. The following reviewer(s) agreed to reveal their identity: Corette Wierenga, Ismael Galve-Roperh.

As you will see, both referees express interest in your work, but raise a number of points that need to be addressed before we can consider publication. In particular both reviewers request a substantial re-write of the text to make your findings easier to understand and clarify important details. They also suggest improvements in the layout and annotation of several of the figures and some additional experiments and quantification of results. Appended below are the points from each reviewer that, following consultation between myself and the reviewers, we have agreed are essential to address:

Reviewer 1:

In this manuscript the authors describe the developmental expression pattern of CB1, DAGLα and MAGL. They also analyze developmental alterations in CB1 KO and WT mice. The data seem solid, and the findings are relevant for cerebellar development. The developmental expression patterns of CB1, DAGL and MAGL in the cerebellum are a valuable contribution to the neuroscience field.

Major concerns:

My main concern with this manuscript is that it is hard to read. The figures are not always clear, badly annotated and not even numbered in the manuscript. The text is full of anatomical terms, which are often not indicated in the figures, which makes it impossible to follow the reasoning of the authors. The manuscript lacks quantification. Finally, the discussion needs to be shortened with focus on the current data.

Minor points:

1. Line 225-234: Is does not become clear (at least not to this reviewer) if developing CFs are CB1 positive or not.

2. Line 284-286: Speculations about possible effects for adult brain seem out of place here.

3. Figure 3: I can clearly appreciate the increase in GAP43 intensity, but the abnormal clustering and distribution of long-range axons is not entirely clear from these images. Perhaps a zoom would work? Images are very small.

4. Line 321: ‘below the PCL and above pWM’ - these structures are not indicated in the figure

5. Line 320-326: The authors refer to many different figures, which is hard to follow. I recommend to make a single figure in which all aspects of GC expression patterns that the authors want to point out are summarized. Please annotate images accordingly.

6. Line 335: Fig 4C is referred to after 4D,E. Please keep numbering of figures logically.

7. Line 351: Please also show the weak staining after P15. I do not see much difference in CB1 staining between P8 and P15 in Fig. 6

8. Line 360: Figure 7 does not have a panel D. It stops at P8 in 7C.

9. It is unclear what I see in panels 7A’, B’ and C’ - is the blue staining RORalpha or Calbindin.

10. Fig 8C: here it would have helped tremendously if the MAGL-positive axons in the DCN and the primary fissure would be pointed out in the figure.

11. Line 378: The authors claim ‘dramatic and developmentally dynamic regional differences’, but they only show 1 region at 1 time point? Either show all data to substantiate this point or rephrase.

12. Line 380-384: This speculation seems more appropriate for the discussion.

13. There is no reference to figure 9A

14. Line 389: Where is the data that shows no difference between sexes?

15. Line 394: CB1 KO mice show a smaller midvermal area, but the developmental trajectory does not appear altered.

16.The discussion needs to be shortened and more focused on the current data. For instance, the discussion about lipid signaling (Line 492-502) seems out of scope. It is not clear to this reviewer why possible differences in spike timing (Line 517-522) are relevant for the current manuscript. I do not understand the link to the current results. There is a large discussion/review on how cerebellar size may affect behavior and diseases, that is not directly related to the current results.

Reviewer 2:

The study consist in the characterization of the expression of some endocannabinoid system elements in the perinatal cerebellum. By mean of immunodetection (CB1, DAGL, MAGL) authors describe the cellular populations expressing CB1 receptors (pontocerebellar axons of mossy and parallel fibers, differentiating GCs, pyramidal tract...). Authors describe the expression of DAGL and MAGL in Purkinje cells and their axons, pointing to PC as the source of the ECB 2AG. One of the most remarkable findings is the existence of an anterio-posterior gradient of CB1 expression. Once the ECS was described in the developing cerebellum authors investigated the impact of CB1 ablation using a complete knockout mice strain. In CB1 ko mice, authors describe the existence of axonal fasciculation deficits, a qualitative observation of increased GAP43 protein associated to growth cones and alterations of axonal SMI32. CB1 receptor deficient mice showed decreased lobe surface area, specifically in anterior lobes (I-III mainly) and fine motor impairment. These findings provide substantial evidence of the potential contribution of cannabinoid signalling to cerebellar development. There are however several concerns and technical aspects that deserve attention prior to acceptance.

Major concerns.

1. Currently there are many conditional CB1 knockout mice available and in order to define the direct contribution of CB1 receptors to cerebellar development it would be much more relevant if this aspect is addressed in a cerebellum specific ko mice. Alternatively, authors could tackle the question using a viral-mediated knockdown/Cre-mediated deletion approach in order to confirm changes the role of cerebellar CB1 receptors in developmental deficits and fine motor impairment. At the very least the authors should discuss the limitations of their approach.

2. The degradation of the endocannabinoid 2AG can be mediated also by ABHD enzymes 6 and 12, please include them in the study.

3. The existence of fine motor impairment in CB1 knockout mice has been previously described by different methods and attributed to changes in other neuronal populations (Diaz-Alonso et al, 2012), mainly subcerebral cortical projection neurons. Please discuss accordingly.

4. Authors should provide quantification of the described increase in GAP43 levels (Fig. 3) and neurofilament SMI-32 “uneven” distribution.

5.Authors describe reduced efficiency of sunflower seed opening task, was there any difference in the total number of seeds or other parameters: motivation, anxiety, sensorimotor deficits in CB1 kos that may interfere with these interesting result?

6. Describing the endocannabinoid system as “machinery” does not appropriately reflect its dynamic expression and the modulatory role of this endogenous signalling system. I suggest to use more accepted nomenclature.

7. Anandamide, the other major endocannabinoid, is not even mentioned along the manuscript. Please improve.

8. Improving figure presentation.

Figure 1. Improve readability by increasing labelling font size and pointing with arrows the CB1 receptor expression and clearly labelling the areas of interest described in the text (panels A-C).

Please include the major relevant cerebellar areas in the low magnification images (Fig. 4D; Fig. 5A, 5D; Fig. 7A,B), and the origin of the inset shown in 4E, 7A, 8D.
